# Arabidopsis MACP2 contributes to autophagy induction by modulating starvation-induced reactive oxygen species homeostasis

**DOI:** 10.1007/s44307-025-00078-4

**Published:** 2025-08-20

**Authors:** Ying Zhou, Xue Zhang, Tiancong Qi, Zi-Han Wang, Yao Wang, Lin-Na Wang, Yong-Lun Zeng, Hanjie He, Liwen Jiang, Daoxin Xie, Shi Xiao, Lu-Jun Yu, Qin-Fang Chen

**Affiliations:** 1 State Key Laboratory of Biocontrol, Guangdong Provincial Key Laboratory of Plant Stress Biology, Innovation Center for Evolutionary Synthetic Biology, School of Life Sciences, Sun Yat-Sen University, Guangzhou, 510275 China; 2 Tsinghua-Peking Center for Life Sciences, MOE Key Laboratory of Bioinformatics, School of Life Sciences, Tsinghua University, Beijing, 100084 China; 3 College of Life Science and Technology, Central South University of Forestry and Technology, Changsha, 410004 China; 4 School of Life Sciences, Centre for Cell & Developmental Biology, State Key Laboratory of Agrobiotechnology, The Chinese University of Hong Kong, New Territories, Shatin, Hong Kong, China

**Keywords:** Autophagy, MACP2, ROS, Arabidopsis

## Abstract

In plants, autophagy is a conserved recycling system essential for development and stress responses by targeting cellular components for massive degradation in the vacuole. Our previous work suggested that autophagy contributes to Arabidopsis (*Arabidopsis thaliana*) stress responses by modulating NADPH-oxidase-mediated reactive oxygen species (ROS) homeostasis; however, the molecular link between extracellular ROS and autophagy remains unknown. We performed a yeast two-hybrid screen to identify components involved in autophagy, using the central autophagy component ATG8e as a bait. We identified MEMBRANE ATTACK COMPLEX/PERFORIN-LIKE 2 (MACP2) as an interactor of ATG8e via its the ATG8-interacting motif and confirmed this interaction by co-immunoprecipitation and bimolecular fluorescence complementation assays. *MACP2-*overexpressing lines showed enhanced sensitivity to nutritional starvation, accelerated leaf senescence, and increased hydrogen peroxide (H_2_O_2_) levels, resembling the phenotypes of *atg* mutants defective in autophagy. Conversely, *macp2* knockouts exhibited diminished starvation-induced H_2_O_2_ accumulation and attenuated autophagosome formation and fully suppressed the starvation-hypersensitive phenotypes of the *atg5-1* mutant. In particular, MACP2 was degraded through the autophagy machinery during prolonged starvation, suggesting a feedback regulatory mechanism for maintaining MACP2 homeostasis. Our findings suggest that MACP2 acts as a key regulator in autophagy induction by controlling influx of extracellular H_2_O_2_ in Arabidopsis.

**Supplementary Information:**

The online version contains supplementary material available at 10.1007/s44307-025-00078-4.

## Introduction

In plants, autophagy is an evolutionarily conserved mechanism that modulates responses to environmental stresses and development cues by targeting intracellular components for degradation (Bassham et al. 2006; Huang et al. 2019; Qi et al. 2021; 2023). The hallmark of autophagy is the formation of a double-membrane vesicle called the autophagosome, which facilitates the degradation of enclosed cargoes through the resident acid hydrolases of vacuoles (Liu & Bassham 2012; Zhuang et al. 2018; Qi et al. 2021; 2023). Several autophagy-related proteins (ATGs) have been shown to play indispensable roles at different stages of autophagosome formation via the assembly of four protein complexes: the ATG1–ATG13 protein kinase complex, the ATG6–phosphatidylinositol 3-kinase (PI3K) complex, the ATG9 membrane delivery complex, and the ATG5–ATG12 and ATG8–phosphatidylethanolamine (PE) complexes (Li & Vierstra 2012; Qi et al. 2021; 2023; Huang & Guo 2024). Among ATGs, the ubiquitin-like conjugation protein ATG8 is a key regulator in mediating selective recruitment of cargoes to the autophagosome (Kellner et al. 2017). During selective autophagy, receptors interact with ATG8 through specific ATG8-interacting motif (AIM) or LIR-interacting motifs, characterized by the consensus amino acid sequence W/F/Y-X-X-L/I/V (Liu & Bassham 2012; Marshall et al. 2019; Acheampong et al. 2020). Upon autophagy induction, ATG8 also interacts with several ATGs, such as ATG1 (Nakatogawa et al. 2012), ATG4s (Yoshimoto et al. 2004), ATG6, and ATG7 (Kaufmann et al. 2014; Kaufmann & Wollert 2014), for either cargo recruitment or self-maintenance.

Reactive oxygen species (ROS) are pivotal signaling molecules during plant interactions with their environment (Mittler et al. 2022). As they are by-products of cellular metabolic pathways, the reactivity of ROS is a double-edged sword; at low levels, ROS function as key secondary messengers to maintain plant growth and development, while the excessive accumulation of ROS poses a substantial threat to cellular macromolecules by causing their irreversible oxidation (Halliwell 2007; Castro et al. 2021). Moreover, ROS production in different subcellular compartments, including the apoplast and intracellular organelles, initiates specific oxidative signaling pathways in plant cells (Castro et al. 2021).

Increasing evidence suggests that autophagy modulates cellular ROS homeostasis *in planta* (Chen et al. 2015a; Huang et al. 2019; Qi et al. 2021). ROS accumulation inside cells is responsible for the oxidative damage of proteins, lipids, or even organelles, which activates autophagy for their clearance (Xiong et al. 2007; Qi et al. 2021). In particular, autophagy distinguishes itself by its capacity to eliminate entire mitochondria and peroxisomes, two ROS-generating organelles, thereby limiting intracellular ROS production (Zhou et al. 2022). By contrast, apoplastic ROS are primarily produced by plasma membrane–localized flavin-containing NADPH oxidases, with the NADPH oxidases RESPIRATORY BURST OXIDASE HOMOLOG D (RBOHD) and RBOHF being major players in apoplast ROS production (Castro et al. 2021). Previous studies have suggested that RBOHs are required in the leaves and roots of Arabidopsis (*Arabidopsis thaliana*) plants for autophagosome biogenesis in response to hypoxia and for plant tolerance to submergence or waterlogging (Chen et al. 2015a; Guan et al. 2019). Thus, apoplastic ROS likely contribute to the regulation of stress responses through the functionality of autophagy. However, the molecular link between extracellular ROS and autophagy induction is still unknown.

Members of the MEMBRANE ATTACK COMPLEX AND PERFORIN (MACP) family are major regulators during pathogen infections and the microbe-associated molecular pattern (MAMP)-triggered immunity in a variety of organisms (Rosado et al. 2008; Fukunaga et al. 2017). A signature motif, comprising the sequence Y/S-G-T/S–H-X7-G-G (X), has been identified in MACPFs from animals and plants (Ni & Gilbert 2017; Yu et al. 2020; Chen et al. 2021; Holmes et al. 2021). Upon their oligomerization, MACPFs play key roles in animal development and immunity by forming pores in cellular membranes (Lukoyanova et al. 2016; Moreno-Hagelsieb et al. 2017). However, the biological function and molecular mechanism of plant MACPFs are poorly understood. In Arabidopsis, four genes encode proteins with a conserved MACPF motif: *CONSTITUTIVELY ACTIVATED CELL DEATH 1* (*CAD1*), *NECROTIC SPOTTED LESION 1* (*NSL1*), *MACP1*, and *MACP2* (Yu et al. 2020; Zhang et al. 2022). Among these genes, mutation of *CAD1* or *NSL1* constitutively activates plant defense responses and leads to programmed cell death in a salicylic acid-(SA) dependent manner (Morita-Yamamuro et al. 2005; Noutoshi et al. 2006; Tsutsui et al. 2006; Asada et al. 2011; Fukunaga et al. 2017; Chen et al. 2021). MACP2 regulates programmed cell death and participates in bacterial pathogen resistance and necrotrophic fungal pathogen susceptibility (Zhang et al. 2022). These observations suggest that MACPFs are likely key factors in regulating plant defense signaling.

In this study, we performed a yeast two-hybrid screen to uncover the ATG8-interaction network and identified MACP2 as a potential ATG8 interactor in Arabidopsis. We determined that MACP2 modulates extracellular H_2_O_2_-induced autophagosome biogenesis in response to carbon or nitrogen starvation. Under long-term starvation, however, the MACP2–ATG8 association led to MACP2 degradation via autophagy, revealing a maintenance mechanism for MACP2 protein abundance during prolonged nutritional starvation in plant cells.

## Results

### Screening of ATG8-interacting proteins by yeast two-hybrid assay

Previous work suggested that during plant stress responses, autophagy acts as a protective mechanism to maintain the cellular ROS homeostasis (Chen et al. 2015a; Guan et al. 2019). To investigate the molecular link between plant autophagy and stress-induced ROS accumulation, we screened for interactors of ATG8e by conducting a yeast two-hybrid screen. We identified 15 candidate interacting proteins with functions related to the categories of protein kinase, protein transport family, and decarboxylase, to name a few (Table 1). Among the 15 candidates, we noticed the H_2_O_2_-scavenging enzyme CATALASE 3 (CAT3) (Zou et al. 2015) and a receptor-like cytoplasmic kinase (RLCK) from subgroup VI_A2 participating in plant growth and morphogenesis (Valkai et al. 2020). We also obtained ENDOPLASMIC RETICULUM MORPHOLOGY 2 (ERMO2), a Sec23/Sec24 transport family protein involved in endoplasmic reticulum–to–Golgi trafficking (Qu et al. 2014), confirming the connection between autophagy and endomembrane systems (Xia et al. 2020). In particular, the protein MACP2 caught our attention because it regulates programmed cell death and participates in bacterial pathogen resistance and necrotrophic fungal pathogen susceptibility (Zhang et al. 2022). Moreover, *MACP2* is widely expressed in various plant tissues and its transcript levels are upregulated in response to several biotic and abiotic stresses (Yu et al. 2020). Thus, we chose MACP2 to explore the significance of its association with ATG8.

**Table 1:** Screening of ATG8-interacting proteins by Y2H assay

Gene locus	Description	Name	AIM (xLIR)	PSSM Score
*AT1G03130*	Photosystem I reaction center subunit II	*PsaD-2*	EEFYVI	10
*AT5G45775*	Ribosomal L5P family protein		KEYELL	13
*AT1G01970*	Tetratricopeptide repeat (TPR)-like superfamily protein		ADWLSI RDYTKI RVFDAV	21 16 7
*AT4G04320*	Malonyl-CoA decarboxylase family protein		RVYDVV PTYEVL	9 13
*AT3G07100*	Sec23/Sec24 protein transport family protein	*ERMO2*	EGFLLL	11
*AT2G18890*	Protein kinase superfamily protein	*RLCK VI_A2*	FSFQEI	12
*AT5G28840*	GDP-D-mannose 3',5'-epimerase	*GME*	REFTFI	12
*AT4G20330*	Transcription initiation factor TFIIE, beta subunit		AVFDSL	7
*AT1G54040*	Epithiospecifier protein	*ESP*	RSYDTV SEWTFL LVWEKL	13 20 13
*AT5G46250*	RNA-binding protein	*LARP6a*	KGFVPI SSFLVV	11 11
*AT4G24290*	MAC/Perforin domain-containing protein	*MACP2*	GVFISL	6
*AT1G20620*	Catalase	*CAT3*	FDFDPL	16
*AT4G21110*	G10 family protein		EGWELI	19
*AT3G26650*	glyceraldehyde 3-phosphate dehydrogenase A subunit	*GAPA*	DEFVSI	15
*AT1G07470*	Transcription factor IIA, alpha/beta subunit		AVYIHV	6

PSSM score represents the relative frequency at which a specific residue appears at that position

### MACP2 interacts with ATG8e via the AIM

**Fig. 1:**
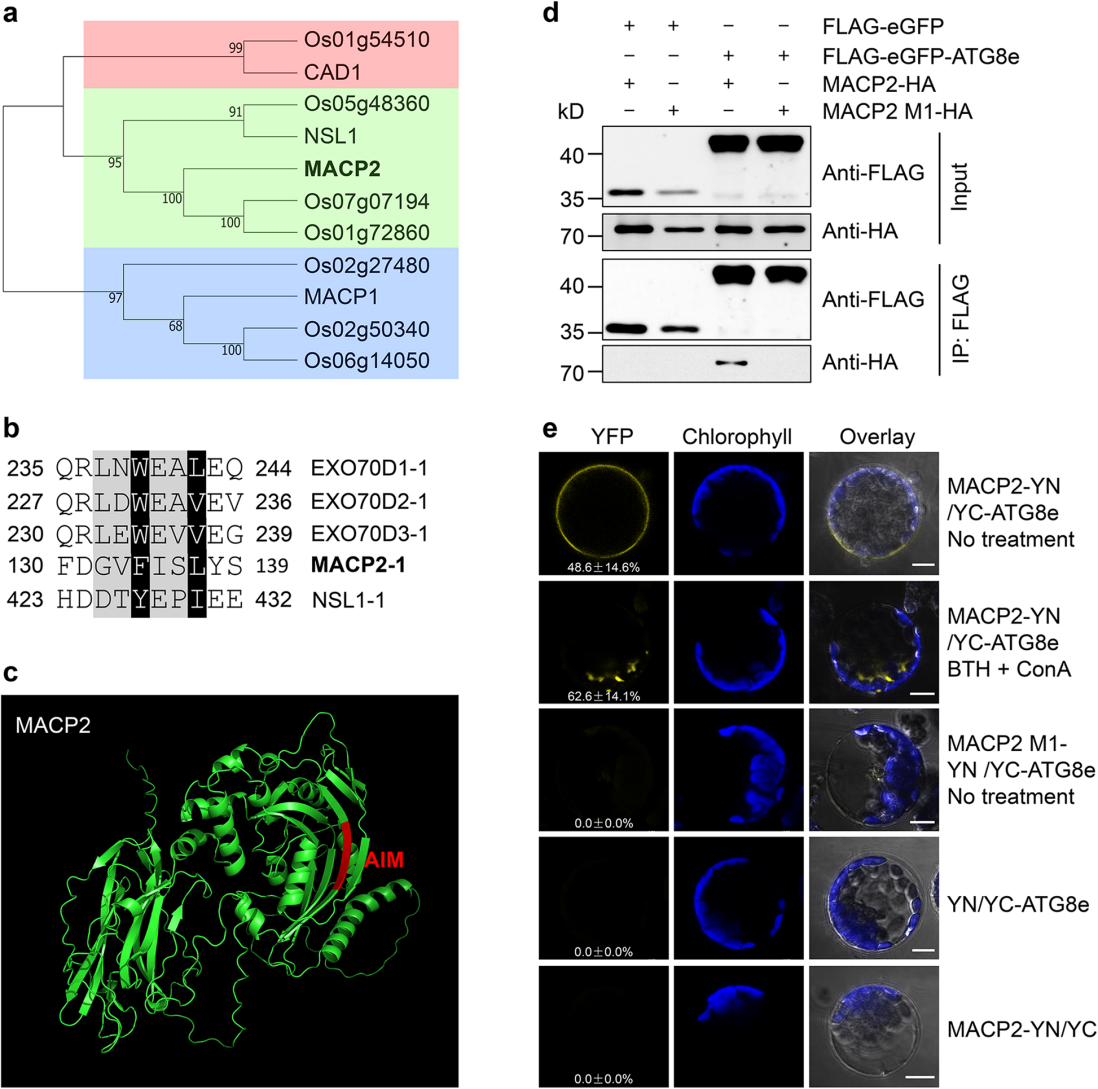
Identification of MACP2 as an ATG8e interactor. **a** Phylogenetic tree of MACPF proteins in Arabidopsis (*Arabidopsis thaliana*) and rice (*Oryza sativa*)*.* The phylogenetic tree was reconstructed via the MEGA X software package with the maximum likelihood method based on the MACPF domain. **b** Sequence alignment of the conserved ATG8-interacting motif (AIM) in MACP2, NLS1, and EXO70Ds of Arabidopsis. EXO70Ds are confirmed ATG8-interacting proteins with AIM. The core AIM is indicated by a gray background. Conserved amino acids in AIM are shown with black backgrounds. Numbers indicate the corresponding amino acid positions. **c** Structure of the AIM in MACP2. AIM located within the MACP2 was analyzed by iLIR database, the protein structure was predicted by Alphafold and visualized using PyMOL software. The AIM is shown in red. **d** Co-immunoprecipitation (Co-IP) assay showing the interaction between ATG8e and MACP2 or MACP2 M1. The constructs *MACP2-HA*, *MACP2 M1-HA (MACP2*^*F134A*,*L137A*^*-HA*), and *FLAG-eGFP-ATG8e* were transfected into mesophyll protoplasts isolated from wild-type Arabidopsis and immunoprecipitated with FLAG affinity magnetic beads. **e** Bimolecular fluorescence complementation (BiFC) assay showing the interaction between MACP2 and ATG8e in vivo. The constructs *MACP2-nYFP* or *MACP2 M1-nYFP* (*MACP2*^*F134A,L137A*^*-nYFP*) were co-transfected with *cYFP-ATG8e* in protoplasts and incubated for 12 h under continuous light conditions, which was followed by treatment with 100 μM benzo [1,2,3] thiadiazole-7-carbothioic acid S-methyl ester (BTH) and 1 μM concanamycin A (ConA) for 6 h. MACP2 M1, MACP2 with F134A and L137A mutations in the AIM. The construct pairs *MACP2-nYFP* + *cYFP* and *nYFP* + *cYFP-ATG8e* were similarly co-transfected into Arabidopsis protoplasts as negative controls. Confocal images for YFP, chlorophyll autofluorescence, and bright field are shown. The numbers in the image indicated the ratio of YFP signal (the number of YFP signal to total protoplasts). The percentages are means ± SD (*n* = 3) calculated from three independent replicates. For each experiment, 15 images were used for the calculation for each coexpression combination. Scale bars, 10 μm

To explore the potential relationship between MACP2 and ATG8, we performed a phylogenetic analysis of MACP2 and related proteins and examined its protein structure and sequence. In Arabidopsis and rice (*Oryza sativa*), MACP members clustered into three groups based on the phylogenetic tree topology (Fig. 1a). Among them, MACP2 is closely related to NSL1 in the second group of the MACP family. A comparison of the MACP2 sequence to those of EXO70Ds (Acheampong et al. 2020) revealed a characteristic xLIR (G132–L137) domain, representing the primary ATG8-interacting motif (AIM), located within the MACPF domain of MACP2 and NSL1, as determined by the iLIR database (Fig. 1b, Fig. S1). Moreover, structural predictions confirmed the presence of an xLIR domain within a β-pleated sheet in MACP2 and an α-helix in NSL1 (Fig. 1c, Fig. S1). By contrast, the other two MACPs of Arabidopsis, MACP1 and CAD1, lacked this AIM (Fig. S1).

To test the interaction between MACP2 and ATG8e *in planta*, we performed co-immunoprecipitation (Co-IP) and bimolecular fluorescence complementation (BiFC) assays. To this end, we co-transfected mesophyll protoplasts prepared from wild-type Arabidopsis plants with the constructs *MACP2-HA* (encoding MACP2 with a HA tag) and *FLAG-eGFP-ATG8e* (encoding a fusion between the green fluorescent protein [eGFP] and ATG8e, together with a FLAG tag) and incubated the transfected cells in darkness for 16 h. When we precipitated ATG8e with anti-FLAG antibodies, we detected MACP2-HA among the immunoprecipitated proteins. To determine the dependence of MACP2-ATG8e interaction on the AIM domain of MACP2, we generated a point mutant form of MACP2 in which the conserved Phe-134 (F134) and Leu-137 (L137) residues were changed to Ala (MACP2^F134A, L137A^, M1) to disrupt the AIM domain. As expected, when we co-transfected the constructs *MACP2 M1-HA* and *FLAG-eGFP-ATG8e* in protoplast cells, FLAG-eGFP-ATG8e did not co-precipitate with MACP2 M1-HA (Fig. 1d). As negative controls, FLAG-eGFP did not co-precipitate with MACP2-HA and MACP2 M1-HA (Fig. 1d).

Similarly, BiFC assays showed that after incubation in continuous light for 12 h, protoplasts co-transfected with *MACP2-nYFP* and *cYFP-ATG8e* demonstrated reconstitution of functional YFP at the plasma membrane, indicative of the MACP2–ATG8e interaction (Fig. 1e). By contrast, co-transfection of *MACP2 M1-nYFP* and *cYFP-ATG8e* failed to produce an intact YFP signal (Fig. 1e). Furthermore, after treatment with the autophagy inducer benzo [1,2,3] thiadiazole-7-carbothioic acid S-methyl ester (BTH, salicylic acid agonist) and the inhibitor of vacuole-type ATPase concanamycin A (ConA), which inhibits the degradation of autophagic bodies, leading to their accumulation (Chen et al. 2015a; Qi et al. 2017; 2023), we observed the accumulation of YFP signal in punctate structures (Fig. 1e). As controls, the *nYFP* + *cYFP*-*ATG8e* and *MACP2-nYFP* + *cYFP* combinations did not yield any YFP signal (Fig. 1e). Taken together, these results suggest that MACP2 interacts with ATG8 through its AIM in vivo.

### *MACP2*-overexpressing lines show accelerated leaf senescence and increased sensitivity to nutrient starvation

**Fig. 2:**
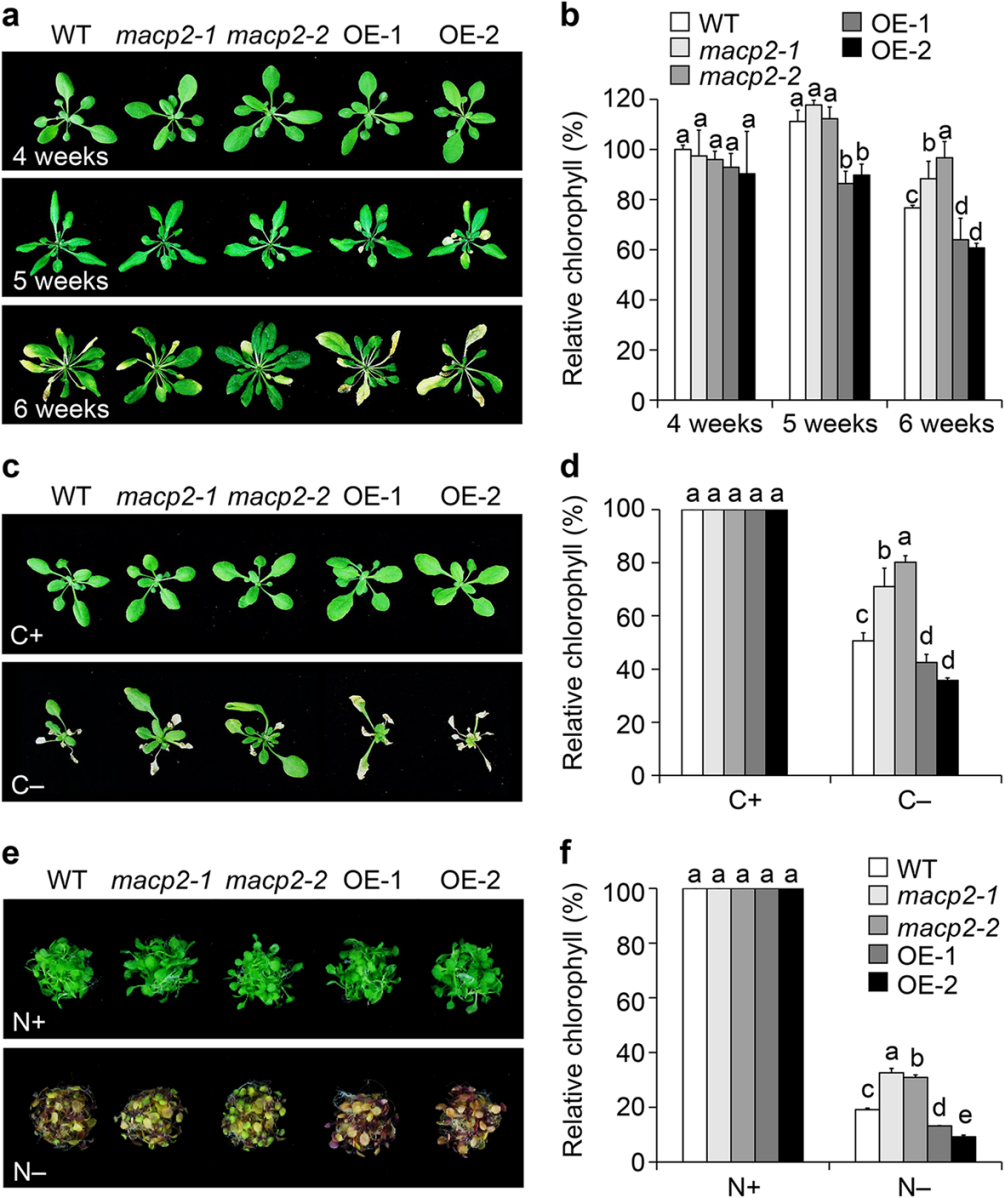
Phenotypes of *macp2* knockout and *MACP2*-overexpressing lines in response to age-dependent senescence and nutrient starvation. **a** and **b** Representative photographs (**a**) and relative chlorophyll contents (**b**) showing the leaf senescence phenotypes of the wild type (WT), *macp2* mutants (*macp2-1* and *macp2*-*2*), and *MACP2-*OE lines (OE-1 and OE-2) grown under a 16-h-light/8-h-dark photoperiod for the indicated number of weeks. Photographs were taken and chlorophyll contents were analyzed at 4, 5, and 6 weeks after germination. For each experiment, three entire plants were used per genotype. The experiments were performed as three biological replicates, each with similar results. Data are means ± SD (*n* = 3) calculated from three biological replicates. Different lowercase letters indicate significant differences within each group as determined by one-way ANOVA via STAT software, *P* < 0.05. **c** and **e** Representative photographs of WT, *macp2* and *MACP2-*OE (OE-1 and OE-2) plants in response to carbon (**c**) or nitrogen starvation (**e**). For carbon starvation, 4-week-old WT, *macp2*, and *MACP2-*OE plants were transferred to continuous darkness (C −) for 7 d and photographed after a 7-d recovery period. For nitrogen starvation, WT, *macp2*, and *MACP2-*OE seedlings grown on half-strength solid Murashige and Skoog (MS) medium for 7 d were transferred to N-rich (N +) or N-free (N −) liquid medium and photographed at 5 d into treatment. **d** and **f** Relative chlorophyll contents of plants in response to carbon starvation (**d**) or nitrogen starvation (**f**) for the indicated times. For each experiment, three entire plants were used per genotype for carbon starvation (**d**) or 20 seedlings were treated per genotype for nitrogen starvation (**f**). All experiments were performed as three biological replicates, each with similar results, and representative data from one replicate are shown. Data are means ± SD (*n* = 3) calculated from three technical replicates. Different lowercase letters indicate significant differences within each group as determined by one-way ANOVA, *P* < 0.05

Previous studies have indicated that the autophagic degradation of chloroplasts occurs during natural premature leaf senescence and in response to nitrogen or carbon deprivation (Wada et al. 2009; Avila-Ospina et al. 2014; Qi et al. 2017; 2023). Conversely, autophagy-defective mutants are characterized by premature leaf senescence and hypersensitivity to nutrient deprivation (Hanaoka et al. 2002; Chung et al. 2010; Qi et al. 2017). Given that MACP2 interacts with ATG8e, we sought genetic evidence to support the potential role of *MACP2* in autophagy. Accordingly, we obtained previously published knockout mutants (*macp2-1* and *macp2-2*) and overexpression lines (*MACP2-*OEs) for *MACP2* (Zhang et al. 2022) for phenotypic analysis. 4-week-old wild-type, *macp2*, and *MACP2-*OE plants did not exhibit leaf senescence phenotypes when grown under normal conditions in a 16-h-light/8-h-dark photoperiod (Fig. 2a). Notably, the cotyledons and several true leaves of *MACP2-*OE plants turned yellow in 5-week-old plants, while those in wild-type and *macp2* plants remained green (middle image; Fig. 2a). Moreover, 6-week-old *macp2* and *MACP2-*OE plants displayed delayed and accelerated senescence, respectively, of their true leaves compared to those of wild-type plants (bottom image, Fig. 2a). We confirmed the above findings by measuring the relative chlorophyll contents of all genotypes and we observed no difference in the chlorophyll contents of 4-week-old plants, while those of 6-week-old *macp2* mutants were significantly higher than that of the wild type. Similarly, the chlorophyll contents of 5- and 6-week-old *MACP2-*OE lines were significantly lower than in the wild type at the corresponding stage (Fig. 2b).

To more directly assess the involvement of MACP2 in the autophagy pathway, we subjected the wild type, the *macp2* mutants, and the *MACP2-*OE lines to carbon or nitrogen starvation by transfer into constant darkness or to nitrogen-free growth medium, respectively. We observed that 4-week-old *macp2* and *MACP2-*OE lines transferred to constant darkness for 7 d displayed enhanced and attenuated resistance, respectively, to fixed carbon starvation compared to wild type (Fig. 2c). In agreement, the chlorophyll contents of the *macp2* and *MACP2-*OE lines were significantly higher and lower, respectively, than that of wild-type plants upon carbon starvation (Fig. 2d). As with carbon starvation, 7-d-old *macp2* and *MACP2-*OE seedlings displayed increased and decreased tolerance of nitrogen deprivation, respectively, relative to the wild type when transferred to nitrogen-free Murashige and Skoog (MS) medium for 5 d (Fig. 2e). Again, we confirmed these results by measuring the relative chlorophyll contents of all genotypes (Fig. 2f). Together, these results suggest that MACP2 is likely a positive regulator of autophagy in plants.

### The accelerated senescence of ***MACP2-***OE plants is associated with the salicylic acid and H_2_O_2_ pathways

**Fig. 3:**
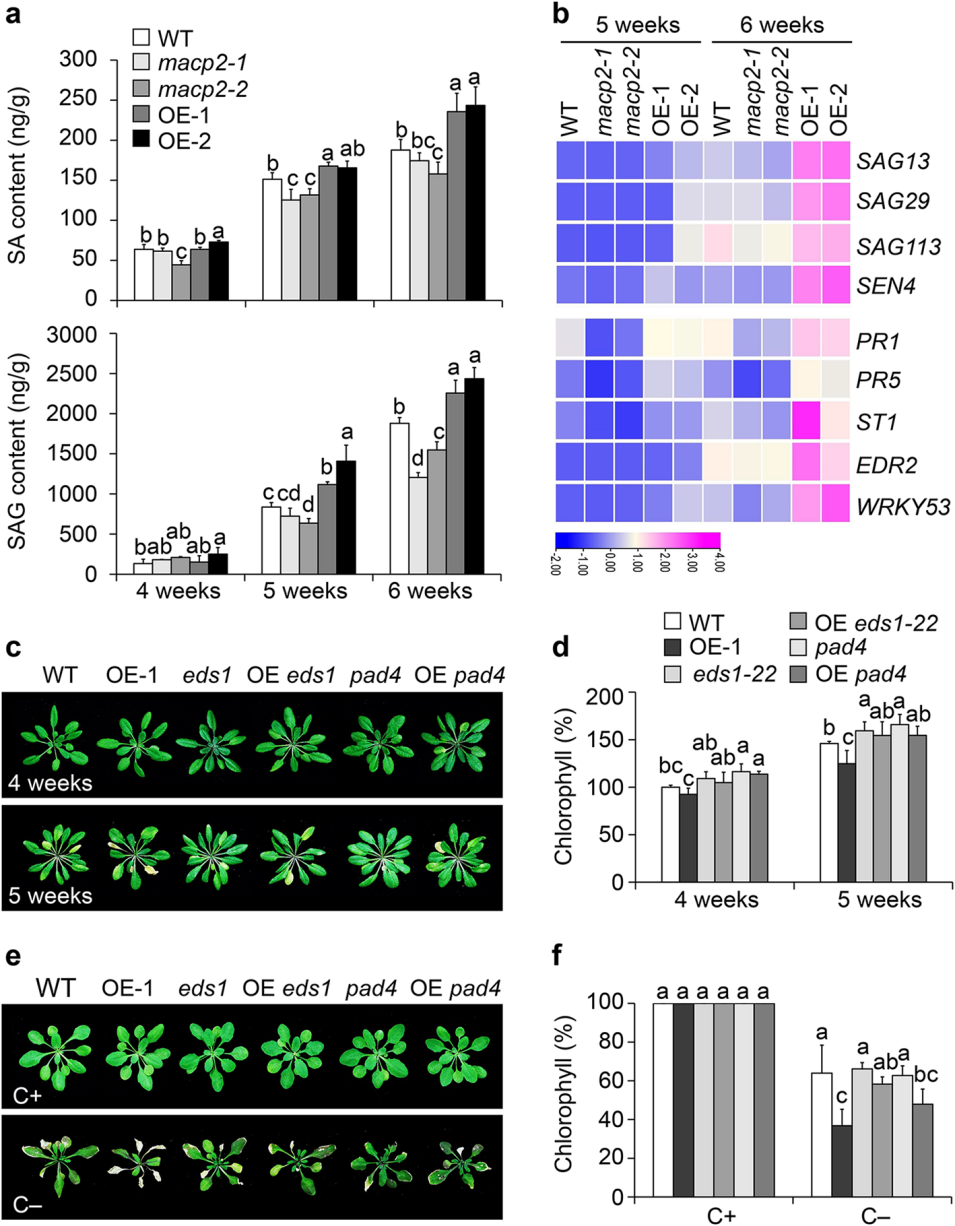
The accelerated senescence phenotypes of *MACP2-*OE plants are associated with salicylic acid pathway. **a** Measurement of salicylic acid (SA) and SA glycoside (SAG) contents in the rosettes of 4-, 5-, and 6-week-old wild type (WT), *macp2*, and *MACP2-*OE (OE-1 and OE-2) plants. The experiments were performed as three biological replicates, each with similar results, and representative data from one replicate are shown. Data are means ± SD (*n* = 3) calculated from three technical replicates. Different lowercase letters indicate significant differences within each group as determined by one-way ANOVA, *P* < 0.05.** b** Hierarchical cluster analysis applied to the 4 DEGs (more than onefold and false discovery rate < 0.05) representing senescence-related genes, and 5 DEGs in the SA pathway by comparing 5- and 6-week-old *macp2* and *MACP2-OEs* rosettes to that of the WT. The transcriptional profiles of relative gene expression values were analyzed using the TB tools command in R. Red and blue represent upregulated and downregulated genes, respectively. **c** Representative photographs showing the leaf senescence phenotypes of 4- and 5-week-old WT, *MACP2-*OE-1, *eds1-22*, OE-1 *eds1-22*, *pad4*, and OE-1 *pad4* plants grown under a 16-h-light/8-h-dark photoperiod. Photographs were taken at 4 and 5 weeks after germination. **e** Representative photographs of WT, *MACP2-*OE-1, *eds1-22*, OE-1 *eds1-22*, *pad4*, and OE-1 *pad4* plants in response to carbon starvation. Four-week-old plants were kept in the light/dark normal condition (C +) or transferred to continuous darkness (C −) for 7 d to induce carbon starvation and photographed after a 7-d recovery in a 16-h-light/8-h-dark photoperiod. **d** and** f** Relative chlorophyll contents of wild-type (WT), *MACP2-*OE-1, *eds1-22*, *pad4*, OE-1 *eds1-22*, and OE-1 *pad4* plants upon natural senescence **(d)**, of 4-week-old plants under dark starvation (**f**), expressed as a percentage relative to control plants or seedlings. For each experiment, 3 entire plants (**d** and **f**) were used per genotype. All experiments were performed as three biological replicates, each with similar results. Relative chlorophyll contents are means ± SD (*n* = 3) calculated from three biological replicates. Different lowercase letters indicate significant differences within each group as determined by one-way ANOVA, *P* < 0.05

Autophagy has previously been shown to be essential for the disposal of damaged organelles and the maintenance of cellular ROS homeostasis in plants under stress conditions; these responses are controlled by the SA signaling pathway (Liu & Bassham 2012; Chen et al. 2015a; Guo et al. 2021). Given that SA and ROS accumulate in Arabidopsis *atg* mutants (Yoshimoto et al. 2009), we measured the endogenous contents for SA and SA glycoside (SAG) of 4-, 5-, and 6-week-old wild-type, *macp2*, and *MACP2-*OE plants grown under normal conditions. We observed few significant differences in SA and SAG contents among 4-week-old wild-type, *macp2*, and *MACP2-*OE plants (Fig. 3a). However, in 5- and 6-week-old plants, the SA and SAG contents in *macp2* and *MACP2-*OE plants were significantly lower and higher, respectively, than those in the wild type (Fig. 3a).

To investigate the correlation between SA and the leaf senescence phenotype of *macp2* and *MACP2-*OE plants, we assessed the transcript levels of senescence-associated genes and of SA-associated defense-responsive genes. Consistent with the senescence phenotypes, we detected a significant upregulation in the transcript levels of the senescence-associated genes *SENESCENCE-ASSOCIATED GENE13* (*SAG13*), *SAG29*, *SAG113*, and *SENESCENCE4* (*SEN4*), as well as of the defense genes *PATHOGENESIS-RELATED1* (*PR1*), *PR5*, *SULFURTRANSFERASE1* (*ST1*), *ENHANCED DISEASE RESISTANCE2* (*EDR2*) and *WRKY53* in the *MACP2-*OE lines compared to the wild type (Fig. 3b). By contrast, the transcript levels of *SAG113*, *PR1*, and *EDR2* were lower in the 6-week-old *macp2* lines than in the wild type (Fig. 3b).

To explore the regulation of MACP2-mediated leaf senescence by SA signaling, we obtained the *MACP2*-OE *pad4* (OE *pad4*) and *MACP2*-OE *eds1* (OE *eds1*) lines (Zhang et al. 2022) for phenotypic analysis. Compared to wild-type, *eds1*, and *pad4* plants, the accelerated natural senescence (Fig. 3c) and starvation hypersensitivity (Fig. 3e, Fig. S2a) phenotypes seen in *MACP2*-OE (OE-1) were attenuated by the *eds1* and *pad4* mutations in the OE *eds1* and OE *pad4* lines, which was also reflected in the relative chlorophyll contents of these genotypes (Fig. 3d, f, Fig. S2b). Thus, the accelerated aging senescence seen in *MACP2-*OE plants is dependent on the SA pathway.

**Fig. 4:**
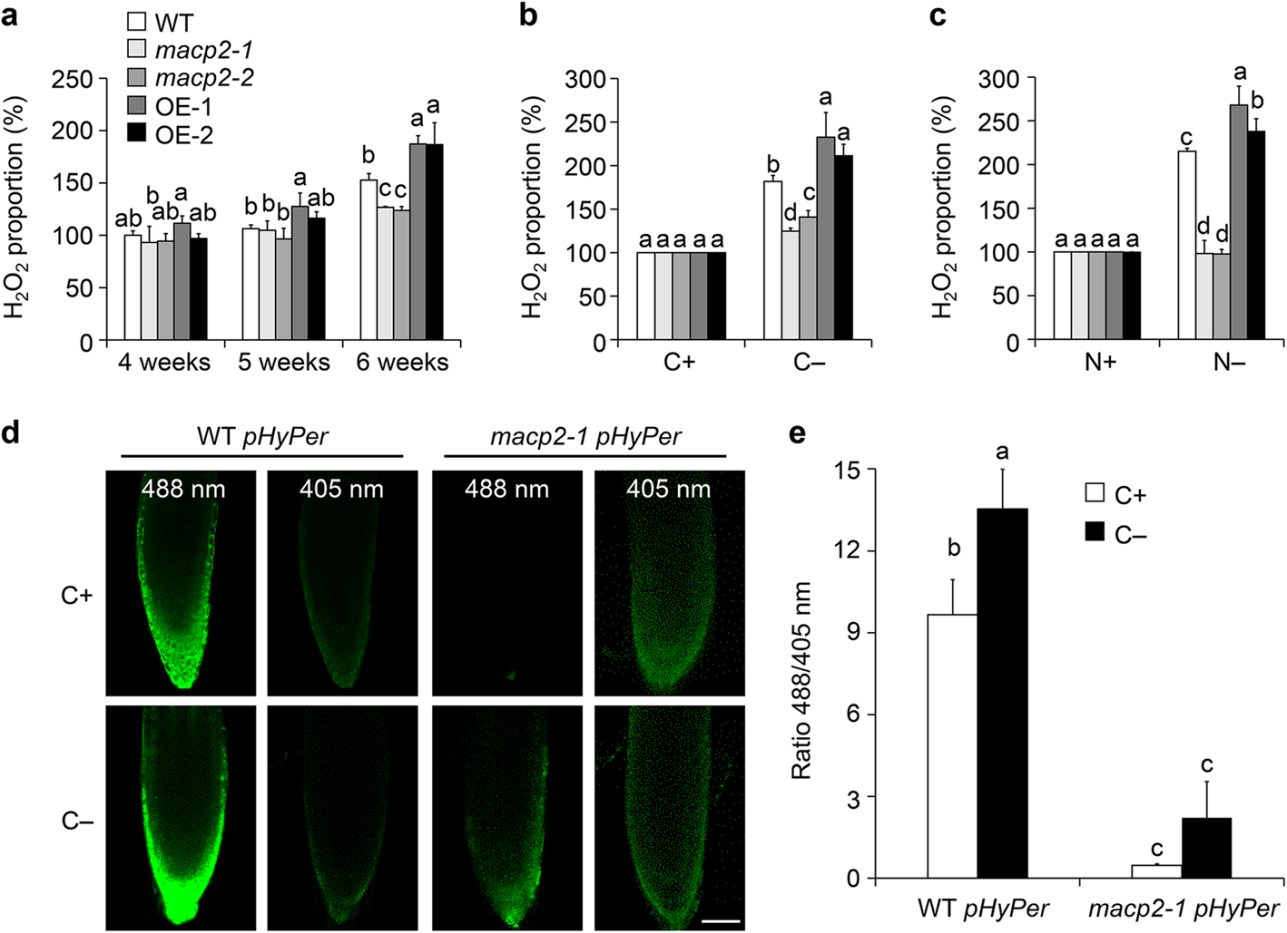
The accelerated senescence phenotypes of *MACP2-*OE plants are associated with H_2_O_2_ influx. **a** to** c** Relative H_2_O_2_ contents of wild-type (WT), *macp2*, and *MACP2-*OE (OE-1 and OE-2) plants grown under a 16-h-light/8-h-dark photoperiod for the indicated number of weeks (**a**), 4-week-old plants with or without a 7-d carbon starvation treatment (**b**), or of 7-d-old seedlings with or without a 7-d nitrogen starvation (**c**) treatment. For each experiment, three entire plants **(a** and **b)** or 20 seedlings (**c**) were used per genotype. All experiments were performed as three biological replicates, each with similar results. Data are means ± SD (*n* = 3) calculated from three biological replicates. Different lowercase letters indicate significant differences within each group as determined by one-way ANOVA, *P* < 0.05. **d** and **e** Arabidopsis plants expressing the *Hyper* molecular probe in WT and *macp2-1* backgrounds to generate transgenic lines (WT *pHyper* and *macp2 pHyper*). The 7-d-old WT *pHyper* and *macp2 pHyper* seedlings were treated with or without a 24-h carbon starvation. Images represent the region of root cells excited at 488 and 405 nm, respectively (**d**). Scale bars, 50 μm. (**e**) Quantification of *pHyPer* fluorescent signals in root cells from the WT *pHyper* and *macp2 pHyper* in (**d**). All experiments were performed as three biological replicates, each with similar results. Data are means ± SD (*n* = 3) calculated from three biological replicates. For each biological replicate, 15 seedlings were used for the calculation for per genotype. Different lowercase letters indicate significant differences between groups as determined by one-way ANOVA, *P* < 0.05

To examine a possible association between the senescence phenotypes and ROS levels in *macp2* and *MACP2-*OE lines, we measured the total hydrogen peroxide (H_2_O_2_) levels in the plants of all genotypes plants during senescence and nutritional starvation using an Amplex Red-based fluorescence assay. The 5- and 6-week-old *MACP2-*OE plants exhibited significantly higher H_2_O_2_ levels than the wild type, while 6-week-old *macp2* mutant plants displayed significantly lower H_2_O_2_ levels when grown under normal conditions (Fig. 4a). Following 7 d of carbon or nitrogen starvation, *macp2* and *MACP2-*OE plants exhibited significantly lower and higher H_2_O_2_ levels, respectively, than the wild type (Fig. 4b, c), suggesting that MACP2 may be involved in maintaining cellular ROS homeostasis in response to senescence and nutrient starvation.

To examine whether the phenotypes of nutrient starvation in *macp2* mutants were associated with intracellular ROS contents, we measured H_2_O_2_ levels in the roots of 7-d-old *pHyPer* transgenic lines in wild type and *macp2* backgrounds (WT *pHyPer*, *macp2 pHyPer*) upon carbon starvation. HyPer is a genetically H_2_O_2_-specific biosensor to be nicely correlated with intracellular H_2_O_2_ changes by determining the ratio of fluorescent signals at an excitation wavelength of 488 nm relative to that at 405 nm (Hernández-Barrera et al. 2013; Shi et al. 2022; Krüger et al. 2021). As shown in Fig. 4, we observed that the ratio of fluorescent signals was enhanced after carbon starvation in the roots of wild type (Fig. 4d, e). Moreover, we showed that the ratio of fluorescent signals in *macp2* was substantially lower than that of wild type before and after carbon starvation (Fig. 4d, e), indicating that the mutation of *MACP2* in plants inhibits the influx of H_2_O_2_ in plant cells.

In plant cells, the excess ROS activates the production of antioxidants, including glutathione (GSH), to eliminate ROS-induced damage and maintain cellular homeostasis (Sharma et al. 2012; Yuan et al. 2017). Given that elevated levels of H_2_O_2_ were detected in *MACP2-*OE plants under nutritional starvation stress, we further investigated the effects of GSH application on the sensitivity of carbon-deficient treatment in wild-type, *MACP2-*OE, and *atg5-1* seedlings. Under normal growth conditions (CK), the *MACP2-*OE (OE-1 and OE-2), and *atg5-1* seedlings exhibited little morphological changes from that of wild type with or without 500 μM GSH application for 12 d. However, the increased sensitivities of *MACP2-*OE plants to the carbon starvation (C −) were completely rescued by supplying GSH in carbon-free solid half-strength MS growth medium (Fig. S3a). As a control, the hyper-sensitive phenotypes of *atg5-1* mutant were also partly restored by 500 μM GSH application (Fig. S3a). These findings were further confirmed by measuring the relative chlorophyll contents of all genotypes, which showed that *MACP2-*OE (OE-1, OE-2) and *atg5-1* plants accumulated significantly less chlorophylls than the wild type upon carbon starvation. With comparison, we did not observe the difference between wild type and *MACP2-*OE lines upon starvation with GSH supplied (Fig. S3b).

### MACP2 is required for starvation-induced autophagosome formation

**Fig. 5:**
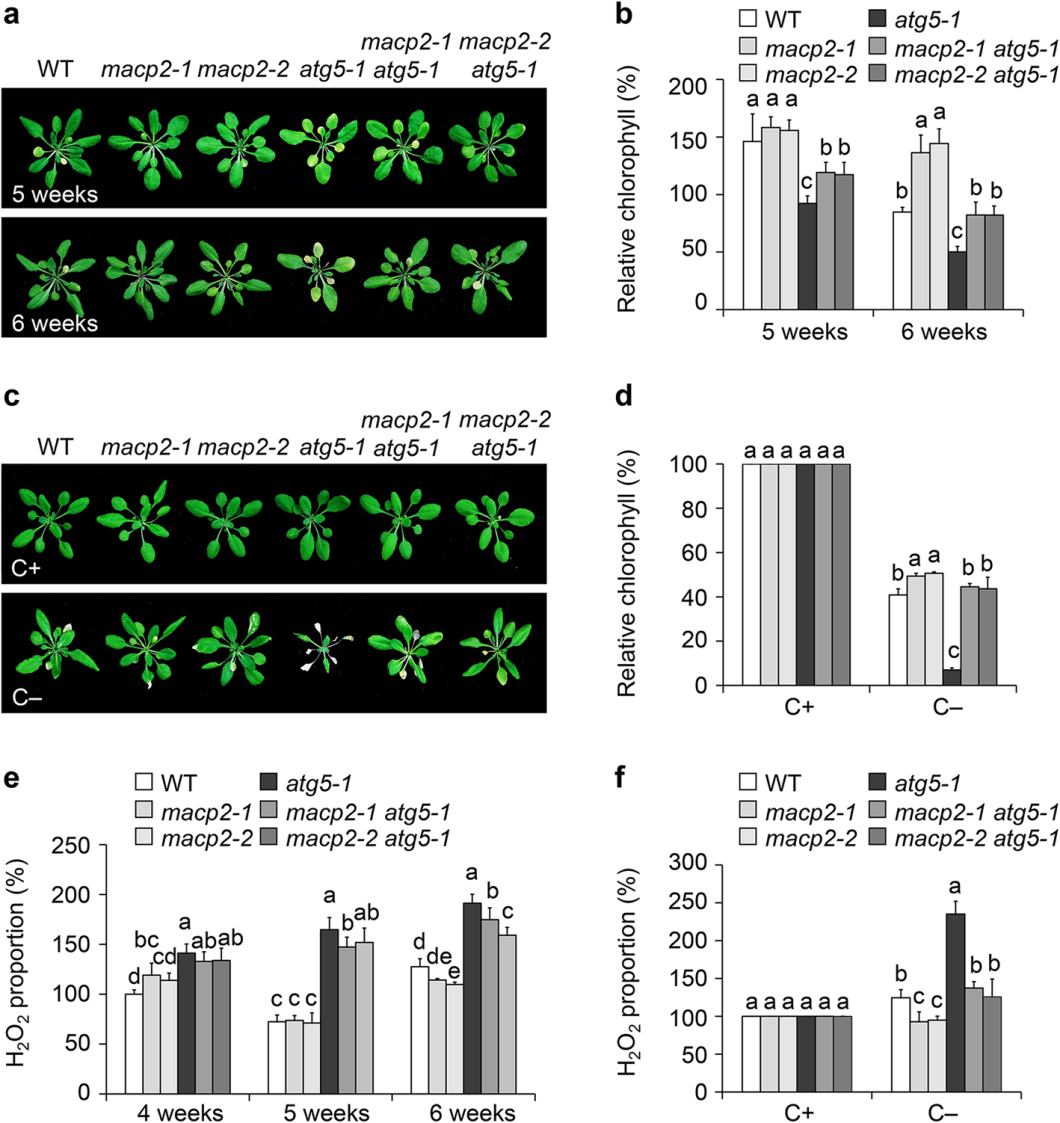
Knockout of *MACP2* rescues the accelerated senescence and ROS accumulation phenotypes of the *atg5* mutant.** a** and** b** Representative photographs (**a**) and relative chlorophyll contents (**b**) showing leaf senescence in the wild type (WT), *macp2*, *atg5-1*, and *macp2* *atg5* double mutants (*macp2-1 atg5-1* and *macp2-2 atg5-1*) grown under a 16-h-light/8-h-dark photoperiod. Photographs and the relative chlorophyll contents were obtained at 5 and 6 weeks after germination. For each experiment, three entire plants were used per genotype. The experiments were performed as three biological replicates, each with similar results, and representative data from one replicate are shown. Data are means ± SD (*n* = 3) calculated from three technical replicates. Different lowercase letters indicate significant differences within each group as determined by one-way ANOVA, *P* < 0.05. **c** and** d** Representative photographs of (**c**) and relative chlorophyll contents (**d**) showing the WT, *macp2*, *atg5-1*, and *macp2* *atg5* double mutants in response to constant darkness treatment. Four-week-old WT, *macp2*, *atg5-1*, and *macp2* *atg5-1* double mutant plants were transferred to continuous darkness for 7 d and photographed after a 7-d recovery in a 16-h-light/8-h-dark photoperiod. **e** and** f** H_2_O_2_ contents in WT, *macp2*, *atg5-1*, and *macp2* *atg5* double mutant plants during senescence (**e**) or following a carbon starvation treatment (**f**). For each experiment (**b** and **d–f**), three entire plants were used per genotype. All experiments were performed as three biological replicates, each with similar results. Relative chlorophyll and H_2_O_2_ contents are average values ± SD (*n* = 3) calculated from three biological replicates. Different lowercase letters indicate significant differences within each group as determined by one-way ANOVA, *P* < 0.05

Given that MACP2 is important for plant senescence and susceptibility to nutrient starvation, we investigated the genetic relationship between MACP2 and the autophagy pathway. To this end, we generated two *macp2 atg5* (*macp2-1 atg5-1* and *macp2-2 atg5-1*) double mutants by crossing each *macp2* mutant to *atg5-1*. As shown in Fig. 5a, compared to wild type, the *macp2* mutants had delayed leaf senescence, while the *atg5-1* mutants showed an accelerated leaf senescence phenotype, confirming previous findings (Liu & Bassham 2012). Importantly, the *macp2 atg5* double mutants displayed a partial rescue of the accelerated senescence phenotype seen in *atg5-1*, with fewer yellow leaves than *atg5-1* at both 5 and 6 weeks (Fig. 5a). We confirmed this observation by quantification of relative chlorophyll contents. The *macp2 atg5* double mutants accumulated significantly more chlorophyll than *atg5-1*, with levels comparable to that of the wild type in 6-week-old plants (Fig. 5b). Similarly, the *atg5-1* mutant was hypersensitive to carbon or nitrogen starvation (Fig. 5c, Fig. S4a), as previously reported (Liu & Bassham 2012). However, 4-week-old *macp2 atg5* plants displayed enhanced tolerance of starvation, with more non-senescing green leaves, resembling the *macp2* mutants (Fig. 5c, Fig. S4a). We confirmed these results by determining relative chlorophyll contents (Fig. 5d, Fig. S4b).

Several autophagy-deficient mutants including *atg5-1* were shown to constitutively accumulate ROS, which contributes to the age- and starvation-dependent promotion of leaf senescence in these mutants (Yoshimoto et al. 2009). Since the *macp2* mutants accumulate lower ROS levels in response to starvation (Fig. 4b, c), we speculated that the recovery of the senescence phenotypes seen in *atg5-1* by *macp2* may be related to H_2_O_2_ contents. To test this possibility, we examined the H_2_O_2_ contents in wild-type, *macp2*, *atg5-1* and *macp2 atg5* plants during natural senescence and upon nutrient starvation. Compared to the *atg5-1* mutant, H_2_O_2_ levels were significantly lower in 5- and 6-week-old *macp2 atg5* double mutant plants experiencing natural senescence (Fig. 5e), as in *macp2 atg5* double mutant plants under fixed carbon starvation for 7 d (Fig. 5f). These results support the idea that the phenotypes observed in the *macp2 atg5* double mutants are due to their reduced ROS production. These findings indicate that MACP2 may regulate autophagy by modulating ROS dynamics in response to age- and starvation-mediated senescence.

**Fig. 6:**
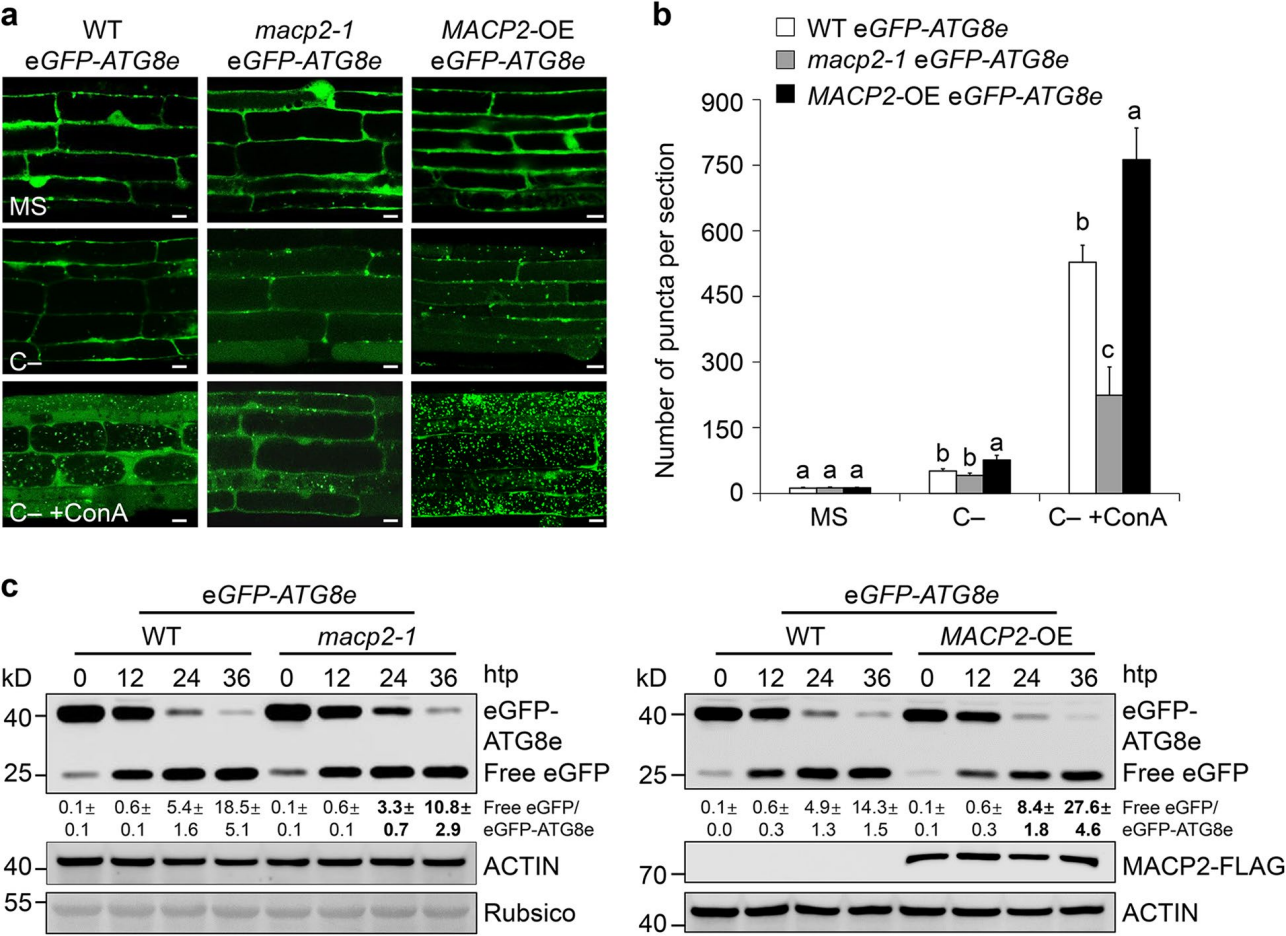
MACP2 modulates carbon starvation–induced autophagosome formation. **a** and** b** Confocal analysis showing eGFP-ATG8e labeled autophagosomes in the root cells of wild-type (WT), *macp2-1*, and *MACP2*-OE seedlings. 7-d-old *eGFP-ATG8e*, *macp2-1 eGFP-ATG8e*, and *MACP2-*OE *eGFP-ATG8e* (*MACP2-FLAG eGFP-ATG8e*) lines were subjected to normal (CK) or carbon starvation (C −) conditions, alone or with 1 µM ConA treatment for 16 h. The formation of autophagosomes was visualized by fluorescence confocal microscopy. Scale bars, 10 μm. (**b**) Number of puncta per root section in root cells from the *eGFP-ATG8e*, *macp2-1 eGFP-ATG8e*, and *MACP2-*OE *eGFP-ATG8e* lines in (**a**). Data are means ± SD (*n* = 3) calculated from three biological replicates. For each experiment, 15 sections were used for calculation per genotype. Different lowercase letters indicate significant differences within each group as determined by one-way ANOVA, *P* < 0.05. **c** Immunoblot analysis showing the processing of eGFP-ATG8e in WT, *macp2-1*, and *MACP2*-OE seedlings in response to carbon starvation (C −). Seven-d-old *eGFP-ATG8e*, *macp2-1 eGFP-ATG8e*, and *MACP2-*OE *eGFP-ATG8e* (*MACP2-FLAG eGFP-ATG8e*) seedlings were collected at 0, 12, 24, or 36 h post transfer (hpt) to constant darkness. Anti-eGFP antibodies were used for immunoblotting. The positions of the eGFP-ATG8e fusion and of free eGFP are indicated at right. The experiments were performed as three biological replicates, each with similar results. Data are means ± SD (*n* = 3) calculated from three biological replicates. The ratio between free eGFP and eGFP-ATG8e is shown below. The numbers at left indicate the molecular weight (kD) of each size marker. Anti-ACTIN antibodies and Ponceau S–stained membranes are shown below the blot to indicate the amount of protein loaded per lane

To further explore the involvement of MACP2 in regulating autophagy, we crossed the *macp2-1* mutant and *MACP2*-OE line to a transgenic line expressing *eGFP-ATG8e*, which is widely used as an autophagosome marker (Xiao et al. 2010; Chen et al. 2015a), to generate the *macp2-1* *eGFP-ATG8e* and *MACP2*-OE *eGFP-ATG8e* lines. We subjected the *eGFP-ATG8e* (*eGFP-ATG8e* in the wild-type background), and *macp2-1* *eGFP-ATG8e* and *MACP2*-OE *eGFP-ATG8e* lines to normal growth conditions or to constant darkness in the presence or absence of 1 μM ConA for 16 h. An examination of fluorescence patterns by confocal microscopy suggested that in the absence of ConA treatment, the numbers of eGFP-ATG8e-labeled autophagic puncta in the root cells of *eGFP-ATG8e* and *macp2-1 eGFP-ATG8e* lines were comparable regardless of fixed carbon starvation (Fig. 6a, b). Upon 12-h carbon starvation, however, the numbers of autophagic puncta in the root cells of *MACP2*-OE *eGFP-ATG8e* was significantly increased than that of *eGFP-ATG8e* cells (Fig. 6a, b). Moreover, the puncta in the *eGFP-ATG8e* line were markedly increased upon carbon starvation in the presence of ConA (C − + ConA; Fig. 6a, b). Notably, starvation-induced autophagic puncta accumulated in the root cells of *macp2-1 eGFP-ATG8e* and *MACP2*-OE *eGFP-ATG8e* were significantly lower and higher, respectively, than that of *eGFP-ATG8e* with ConA application (Fig. 6a, b). As an independent method to measure autophagic flux, we employed the release of free eGFP to monitor the degradation of the eGFP-ATG8e reporter into the vacuole by autophagy (Qi et al. 2017; 2023). Consistent with the above confocal microscopy results, the ratio of free eGFP/eGFP-ATG8e following carbon starvation was lower in *macp2-1 eGFP-ATG8e* and higher in *MACP2*-OE *eGFP-ATG8e* seedlings, compared to the *eGFP-ATG8e* line (Fig. 6c), indicating that MACP2 is required for the starvation-induced formation of autophagosomes in Arabidopsis.

Given that MACP2 influences the production of autophagosomes in plant responses to nutritional starvation, we further investigate the potential role of MACP2 in modulating ROS-induced autophagy biogenesis. To this end, we subjected the *eGFP-ATG8e*, *macp2-1* *eGFP-ATG8e*, and *MACP2*-OE *eGFP-ATG8e* lines to half-strength MS liquid medium with 100 μM H_2_O_2_ and SA agonist BTH (benzo [1,2,3] thiadiazole-7-carbothioic acid S-methyl ester) for the indicated times and then observed by confocal microscopy. We suggested that both treatments induced the formation of eGFP-ATG8e-labbled autophagic puncta significantly compared to the control in WT background (Fig. S5a-d), confirming previous findings (Xiong et al. 2007; Yoshimoto et al. 2009; He et al. 2023). Moreover, we showed that compared to the WT plants, the *macp2-1* mutant and *MACP2*-OE accumulated significant less or more autophagic puncta in response to either H_2_O_2_ or BTH (Fig. S5a-d).

### Autophagy contributes to MACP2 degradation upon long-term stress exposure

**Fig. 7:**
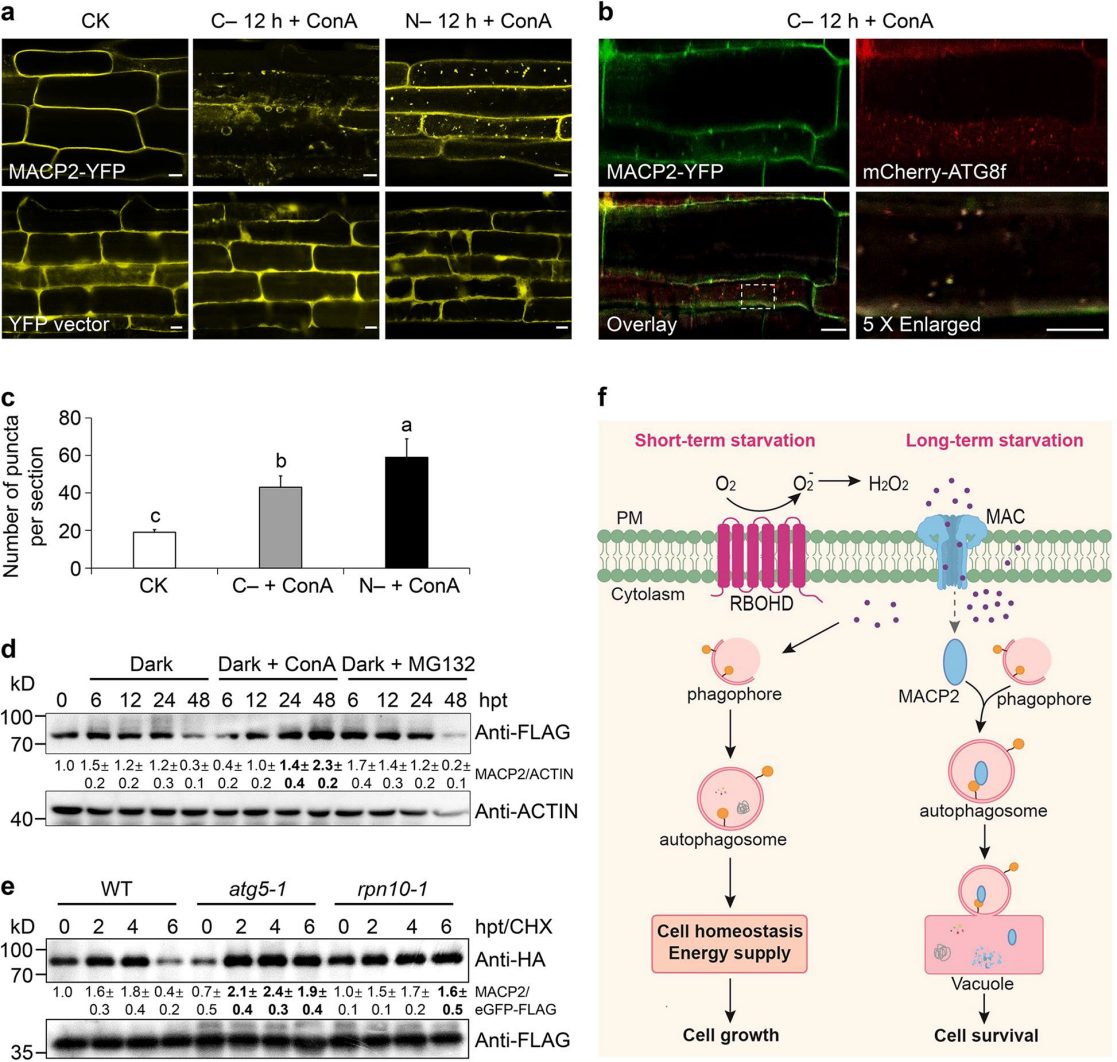
Feedback regulation of MACP2 by autophagy upon long-term stress exposure.** a** and** c** Subcellular localization of MACP2-YFP (upper images) in the root cells of 7-d-old *MACP2-YFP* seedlings under normal growth conditions (CK) or upon 12-h carbon (C − 12 h) or nitrogen starvation (N − 12 h) in the presence of 1 μM ConA. The localization of YFP alone (lower images) under similar conditions was used as a control. Scale bars, 10 μm. (**c**) Number of puncta per root section in root cells from *MACP2-YFP* seedlings under normal growth conditions (CK), 12-h carbon (C − 12 h + ConA) and nitrogen starvation (N − 12 h + ConA) in (**a**). Data are means ± SD (*n* = 3) calculated from three biological replicates. For each experiment, 15 sections were used for calculation per genotype. Different lowercase letters indicate significant differences between groups as determined by one-way ANOVA, *P* < 0.05. **b** Colocalization of MACP2-YFP with mCherry-ATG8f in response to carbon starvation in the presence of ConA (C − 12 h + ConA). The *MACP2-YFP* line was crossed to *mCherry-ATG8f* to generate the double transgenic line *mCherry-ATG8f MACP2-YFP*. Colocalization assay of MACP2-YFP and mCherry-ATG8f in transgenic *mCherry-ATG8f MACP2-YFP* seedlings after treatment with 1 µM ConA under continuous darkness for 12 h is shown. The experiments were performed as three biological replicates, each with similar results. For each experiment, 15 sections were used for observation for per genotype. Scale bars, 10 μm. **d** Treatment with the autophagy inhibitor ConA attenuates the degradation of MACP2. 7-d-old *MACP2-FLAG* transgenic seedlings were exposed to carbon starvation in the presence and absence of 1 μM ConA or 50 μM MG132 for the indicated times (0, 6, 12, 24, or 48 h). hpt, hours post-treatment. The experiments were performed as three biological replicates, each with similar results. The abundance of ACTIN was used as a control. The relative quantification of MACP2-FLAG protein intensity was normalized to the corresponding ACTIN control and then compared to its level at 0 h. Data are means ± SD (*n* = 3) calculated from three biological replicates. **e** Degradation pattern of MACP2-HA in the wild type (WT), *atg5-1*, and *rpn10-1*. The *MACP2-HA* and e*GFP-FLAG* plasmids were co-transfected into protoplasts isolated from WT, *atg5-1*, and *rpn10-1* mutants. After overnight culture, protoplasts were treated with 50 μM cycloheximide (CHX) under darkness for the indicated times (0, 2, 4, or 6 h). The experiments were performed as three biological replicates, each with similar results. The abundance of eGFP-FALG was used as a control. The relative quantification of MACP2-HA protein intensity was normalized to the corresponding eGFP-FALG control and then compared to its level at 0 h. Data are means ± SD (*n* = 3) calculated from three biological replicates. The numbers on the left indicate the molecular weight (kD) of each size marker. hpt, h post-treatment. **f** A working model for MACP2 in the regulation of autophagy induction by controlling influx of extracellular ROS in Arabidopsis. During leaf senescence or in plant response to nutrient deprivation, MACP2 protein forms a homo- or hetero-polymer at the plasma membrane to form a membrane attack complex (MAC) pore, which mediates the transport of the extracellular H_2_O_2_ produced through RBOHs into the cytoplasm. During the early senescence or starvation stages, H_2_O_2_ acts as a signal molecule to stimulate autophagy, which maintains cellular homeostasis and energy supply, ensuring cell growth (left panel). By contrast, the continuous accumulation of H_2_O_2_ triggers the association of MACP2 with autophagosomes and targets MACP2 for its vacuolar degradation (right panel), thus mediating cellular ROS homeostasis and promoting cell survival under long-term starvation conditions

To verify the link between MACP2 and autophagy, we determined the subcellular localization of MACP2 in transgenic lines expressing *MACP2-YFP* (Zhang et al. 2022). Confocal microscopy showed that under normal growth conditions, the MACP2-YFP fusion predominantly localized at the plasma membrane in the root cells of 7-d-old seedlings (Fig. 7a). However, when the seedlings were transferred to constant darkness (C −) or subjected to nitrogen starvation treatment (N −) for 12 h, fluorescent signals for MACP2-YFP appeared to accumulate as vesicles in the cytoplasm (Fig. 7a-c). In the *YFP* control line, we detected YFP signals in the cytoplasm and the nucleus under both normal growth conditions and nutrition starvation (C‒ and N −; Fig. 7a).

To determine the nature of the punctate MACP2-YFP signals, we tested the colocalization of MACP2 with the autophagosome marker ATG8. To this end, we generated a *MACP2-YFP mCherry-ATG8f* double transgenic line by crossing *MACP2-YFP* with *mCherry-ATG8f* encoding a fusion between the red fluorescent protein mCherry and ATG8f (Xia et al. 2020). Indeed, the starvation-induced punctate dots of MACP2-YFP primarily colocalized with mCherry-ATG8f in the root cells of 7-d-old seedlings upon 12 h of carbon or nitrogen starvation in the presence of ConA (Fig. 7b, Fig. S6), suggesting that MACP2 associates with starvation-induced autophagosomes *in planta*.

To explore the potential regulatory mechanism linking autophagy and MACP2, we assayed the stability of MACP2 by using 7-d-old *pUBQ10:MACP2-FLAG* transgenic seedlings (Fig. S7). Immunoblot analysis revealed the accumulation of MACP2-FLAG for the first 24 h following transfer to constant darkness, followed by a marked drop in abundance after 48 h of darkness (Fig. 7d), indicating that MACP2 itself is degraded under long-term stress exposure. When we repeated the experiment in the presence of the proteasome inhibitor MG132 or ConA, which inhibits the degradation of autophagic bodies (Qi et al. 2017, 2023; Xia et al. 2020), we discovered that the application of ConA, but not MG132, suppresses the degradation of MACP2 during darkness for 48 h (Fig. 7d). To verify this finding, we transfected the *MACP2-HA* construct into Arabidopsis protoplasts isolated from the wild type, the autophagy mutant *atg5-1*, and the dual ATG8/Ubiquitin receptor mutant *rpn10-1* defective in REGULATORY PARTICLE NON-ATPASE 10 function (Smalle et al. 2003; Marshall et al. 2015). We then incubated all protoplasts for up to 6 h in the presence of the protein translation inhibitor cycloheximide (CHX; Nazio et al. 2016; 2017). A protein stability assay revealed that MACP2-HA accumulated during the first 4 h of CHX treatment, but decreased after 6 h in continuous darkness in wild-type protoplasts (Fig. 7e). However, this degradation of MACP2-HA was substantially attenuated in the *atg5-1* and *rpn10-1* mutants under the same conditions (Fig. 7e), indicating that the autophagy pathway mediates the degradation of MACP2 under darkness. Furthermore, we investigated the protein abundance of MACP2 and MACP2^F134A,L137A^ variant (MACP2 M1) in wild-type and *atg8e* Arabidopsis protoplasts under darkness for up to 8 h in the presence of CHX. Immunoblot analysis showed the degradation of MACP2-HA was faster in *atg8e* than in wild type. By contrast, the protein abundance of MACP2 M1-HA showed no difference in wild type and *atg8e* (Fig. S8). Taken together, these findings demonstrate that autophagy regulates MACP2 protein stability during darkness starvation exposure.

## Discussion

In plants, autophagy is a degradation pathway that recycles damaged or unwanted cell materials and other macromolecules during development or upon exposure to abiotic and biotic stress (Liu & Bassham 2012; Signorelli et al. 2019). Increasing evidence demonstrates that autophagy and ROS are interconnected; that is, ROS can induce autophagy, and autophagy in turn contributes to lowering excessive ROS accumulation (Signorelli et al. 2019; Zhou et al. 2022). ROS are important early signaling molecules in plant responses to environmental stresses, but excess ROS may cause severe damage to cellular components. Under these circumstances, autophagy is a key mechanism by which ROS levels are kept under control during plant stress responses, possibly by modulating the degradation of ROS-generating organelles (Signorelli et al. 2019; Castro et al. 2021). However, how ROS, and particularly extracellular H_2_O_2_, induce autophagy remains largely unknown to date.

In this study, we present several lines of evidence to support the idea that the membrane attack complex and perforin family protein MACP2 functions as a key regulator of autophagy induction by controlling the influx of extracellular H_2_O_2_ in Arabidopsis. First, MACP2 interacted with ATG8e via its conserved AIM in vivo and translocated from the plasma membrane to autophagosomes in response to nutrient starvation (Fig. 1, 7). Second, knockout of *MACP2* (*macp2*) enhanced plant tolerance to nutrient starvation and delayed leaf senescence. Conversely, *MACP2*-overexpressing lines (*MACP2-*OEs) showed increased sensitivity to nutrient starvation and accelerated leaf senescence (Fig. 2, 3, and 5). Third, *macp2* and *MACP2-*OEs lines showed significantly lower or higher SA and H_2_O_2_ levels, respectively, than wild-type plants during natural age-dependent senescence or in response to nutrient starvation in an SA-dependent manner (Fig. 3, 4). Fourth, upon nutrient starvation, autophagosome formation was compromised in *macp2* compared to wild type (Fig. 6). Finally, MACP2 was degraded through the autophagy machinery during prolonged nutrient starvation (Fig. 7). Thus, our findings demonstrate that MACP2 is involved in autophagy induction by mediating the influx of extracellular ROS, whereas MACP2 itself is feedback regulated by autophagy to control a proper cellular ROS balance.

In animal cells, MACPFs form oligomeric rings in target membranes during cell development and immunity (Lukoyanova et al. 2016; Moreno-Hagelsieb et al. 2017). We recently suggested that in plants, *MACP2* is widely expressed in various plant tissues, and its expression is upregulated in response to biotic and abiotic stresses (Yu et al. 2020). Consistent with this notion, recent findings indicate that MACP2 induces programmed cell death essential for plant susceptibility to bacterial and necrotrophic fungal pathogens (Zhang et al. 2022). Moreover, our findings revealed that the conserved residues in the AIM of MACP2 link its function to autophagy induction by interacting with ATG8 (Fig. 1), suggesting that MACP2 is likely an important upstream regulator of the autophagy pathway involved in diverse stresses responses in plants.

Autophagy is crucial for the maintenance of cellular homeostasis, acting as a quality control mechanism under both normal and stress conditions in plant cells (Qi et al. 2023). Previous studies have shown that H_2_O_2_ and the ROS-producing agent methylviologen (MV) trigger autophagy, the transcript level of *AtATG18a* increased after MV treatment in wild-type seedlings (Scherz-Shouval et al. 2007; Shin et al. 2009; Xiong et al. 2007; Signorelli et al. 2019; Lin et al. 2021). In yeast (*Saccharomyces cerevisiae*) and animal cells, ROS activate autophagy by directly regulating the stability of ATG4 protein (Scherz-Shouval et al. 2007; Essick & Flora 2010; Lin et al. 2021). Therefore, we conceive that ROS-induced autophagy in plants may be mediated through either transcriptional upregulation of core autophagy-related genes, such as *AtATG18a*, or post-translational regulation of ATG proteins stability, as observed in yeast and animals, although the underlying mechanism remains to be elucidated. In Arabidopsis, most autophagy-defective mutants display conventional phenotypes with ROS accumulation, early leaf senescence, and increased sensitivity to nutrient deprivation (Liu & Bassham 2012; Signorelli et al. 2019). Similarly, we observed that *macp2* mutants displayed enhanced tolerance of nutrient starvation and delayed leaf senescence with lower ROS accumulation. By contrast, the *MACP2*-OE lines displayed increased sensitivity to nutrient starvation and accelerated leaf senescence with higher ROS accumulation (Fig. 2–4), suggesting that MACP2 is likely a positive regulator in ROS production in planta. It is worthy to note that like the *MACP2*-OE lines, the *atg* mutants also displayed accelerated senescence phenotypes (Hanaoka et al. 2002; Yoshimoto et al. 2009; Qi et al. 2017), which is likely due to their cellular accumulation of ROS (Signorelli et al. 2019), supporting that ROS is a double-edged sword in autophagy induction and plant tolerance to starvation. Notably, the accelerated natural and starvation-induced senescence phenotypes of *atg5-1* were partially rescued by mutation of *MACP2*, which was also correlated with ROS levels *in planta* (Fig. 5), suggesting that the cellular ROS level is a key determinant factor for induction of the accelerated senescence phenotypes in both the *MACP2*-OE lines and *atg5-1* mutants. Consistent with this notion, the knockout of *MACP2* blocks the intracellular ROS accumulation upon starvation or during senescence, which therefore attenuates its enhanced senescent phenotypes.

RBOHD and RBOHF are two major NADPH oxidases localizing at the plasma membrane to catalyze the production of apoplastic ROS in plant cells (Castro et al. 2021). We and other groups have previously observed that in Arabidopsis, the autophagosome-like structures decrease in the *rbohd*, *rbohf*, and *rbohd rbohf* mutants in comparison to the wild type under hypoxia stress (Chen et al. 2015a; Guan et al. 2019), supporting the role of RBOH-generated ROS in inducing autophagy in plant cells. Given that *macp2* accumulates less H_2_O_2_ than the wild type under natural senescence and nutrient starvation conditions (Fig. 4), it is likely that the diminished H_2_O_2_ influx in the *macp2* mutant contributes to its rescue of the *atg5-1* phenotypes (Fig. 5e, f). Previous works suggest that membrane attack complex (MAC) allows the free passage of water, ions (e.g. Ca^2+^), and/or other molecules, including small proteins across membranes to induce cell death (Morgan 1989; Bakopoulos et al. 2019). We speculate that, in response to environmental cues, MACP2 forms a homo- or hetero-polymer at the plasma membrane to mediate influx of extracellular ROS, which act as signals to stimulate the initiation of autophagosome formation. Consistent with our findings, a recently study shows that the mitochondrial GSDMD (pore-forming protein gasdermin D) protein mediates pore formation to release mitochondrial ROS across the membrane (Weindel et al. 2022). Thus, it is likely that the pore-forming proteins play an important role in mediating the transport of H_2_O_2_ across the membrane in cells. Consistent with our findings, several previous studies reveal that multiple plant aquaporins facilitate H_2_O_2_ transport (Waszczak et al. 2018; Castro et al. 2021). For instance, Arabidopsis PIP1;4 (plasma membrane intrinsic protein 1;4) and PIP2;1 aquaporins mediate H_2_O_2_ influx into the cytoplasm during plant immunity and stomatal closure, respectively (Grondin et al. 2015; Tian et al. 2016). Intriguingly, recent findings reveal that autophagy is involved in the degradation of PIP2;7 under drought stress conditions (Li et al. 2020; Tang & Bassham. 2022), indicating autophagy may function with several membrane-localized proteins modulating cellular ROS trafficking in response to biotic and abiotic stresses. Interestingly, another MACP family member NSL1 also contains the conserved AIM domain (Fig. S1), which is involved in modulating ROS production in plant immunity response (Fukunaga et al. 2017). However, whether NSL1 shares a redundant function with MACP2 in maintaining cellular ROS homeostasis and regulating autophagy biogenesis remain to be further investigated.

Moreover, several studies have revealed that autophagy likely mediates the maintenance of ROS homeostasis under stresses via the NONEXPRESSER OF PR GENES1 (NPR1)–PHYTOALEXIN-DEFICIENT4 (PAD4)–ENHANCED DISEASE SUSCEPTIBILITY1 (EDS1) SA pathway (Liu & Bassham 2012; Chen et al. 2015a; Qi et al. 2017; Guo et al. 2021). Previous studies have demonstrated that SA agonist BTH induces autophagosome formation (Yoshimoto et al. 2009; He et al. 2023; Qi et al. 2023). In Arabidopsis, mutation of *ATG* genes results in upregulation of the SA biosynthesis genes and consequent SA accumulation, thereby influencing both senescence processes and pathogen defense responses (Yoshimoto et al. 2009; Liao & Bassham. 2020). Consistent with this notion, the SA and SAG contents of *macp2* and *MACP2-*OE plants were significantly lower and higher, respectively, than in wild type (Fig. 3a). Moreover, the accelerated natural senescence and hypersensitivity to nutrition starvation seen in *MACP2-*OE lines were completely suppressed in the *eds1* and *pad4* mutant backgrounds (Fig. 3c-f, Fig. S2). Moreover, the SA signaling mutant *npr1* is observed to suppress the early senescence phenotype in *atg5*, suggesting that the senescence phenotype in *atg5* mutant is SA dependent (Yoshimoto et al. 2009). Therefore, the observations that the *macp2* mutation rescued the accelerated senescence phenotype and the sensitivity to nutrient starvation of *atg5-1* in the *macp2 atg5* double mutants (Fig. 5a-d), may be explained by the decreased SA contents in the *macp2* mutants during senescence or upon starvation. Furthermore, previous works have suggested that the accumulation of ROS in *atg* mutants is mediated by SA pathway (Yoshimoto et al. 2009; Chen et al. 2015a). Likewise, the accumulation of ROS can stimulate SA biosynthesis, suggesting these two signaling molecules likely form a positive amplification loop (Shah 2003; Yoshimoto et al. 2009). Collectively, these findings establish that SA and ROS both function as autophagy inducers and mutually reinforcing signaling molecules within an integrated regulatory network. It is worth noting that mutation of either MACPF family gene, *CAD1* or *NSL1*, constitutively activates plant defense responses and programmed cell death in an SA-dependent manner (Morita-Yamamuro et al. 2005; Noutoshi et al. 2006; Tsutsui et al. 2006; Asada et al. 2011; Fukunaga et al. 2017; Chen et al. 2021). Thus, SA is an important regulatory signaling molecule that modulates MACPF function and ROS-triggered autophagy induction.

We further showed that under normal growth conditions, MACP2-YFP predominately localized to the plasma membrane; however, MACP2-YFP translocated to autophagosomes in response to nutrient starvation, as evidenced by the colocalization of MACP2-YFP punctate dots with mCherry-ATG8f-labeled vesicles (Fig. 7a-c, S6). The association of MACP2 with autophagosomes may reflect its feedback degradation by the autophagy machinery under long-term stresses (Fig. 7d, e, Fig. S8), indicating that MACP2 probably regulates and is regulated by the autophagy pathway, which is potentially a key mechanism in controlling a proper cell ROS balance, especially during prolonged starvation exposure. This phenomenon has been frequently observed in the autophagy- and vacuolar sorting–mediated degradation pathways. For instance, the two central protein complexes ATG1–ATG13 and SEVEN IN ABSENTIA OF ARABIDOPSIS (SINATs)–FYVE DOMAIN PROTEIN REQUIRED FOR ENDOSOMAL SORTING1 (FREE1) modulate autophagosome biogenesis and are degraded via the autophagy pathway (Suttangkakul et al. 2011; Xia et al. 2020), thus providing feedback control of autophagy initiation. Notably, our results demonstrated that the MACP2 protein stability is mediated by autophagy pathway, but not through the 26S proteasome pathway during carbon starvation (Fig. 7d, e). However, the degradation of MACP2 was suppressed in the dual ATG8/Ubiquitin receptor mutant *rpn10-1* (Fig. 7e). A previous work demonstrates that the proteasome subunit RPN10 simultaneously binds to both ATG8 and ubiquitin (Marshall et al. 2015). Thus, RPN10 likely acts as a selective autophagy receptor to target inactive 26S proteasomes by concurrent interactions with ubiquitylated proteasome subunits and lipidated ATG8 lining the enveloping autophagic membranes (Marshall et al. 2015). Thus, we considered that RPN10 may mediate the degradation of MACP2 by activation of proteophagy during carbon starvation, rather than proteasome-mediated degradation. However, the precise molecular mechanism underlying RPN10-mediated MACP2 degradation requires further elucidation.

In conclusion, we elucidated the roles of MACP2 in the regulation of autophagy induction through control of the influx of extracellular ROS in Arabidopsis (Fig. 7f). During leaf senescence or in response to nutrient deprivation, MACP2 forms a homo- or hetero-polymer at the plasma membrane to form a membrane attack complex (MAC) pore, which mediates the transport of the extracellular H_2_O_2_ produced through RBOHs into the cytoplasm. During early senescence or nutrient starvation, H_2_O_2_ acts as a signal molecule to stimulate the induction of autophagy, which maintains cellular homeostasis and energy supply, ensuring cell growth (left panel, Fig. 7f). By contrast, the continuous accumulation of H_2_O_2_ triggers the association of MACP2 with autophagosomes and targets MACP2 for its vacuolar degradation (right panel, Fig. 7f), thus mediating cell ROS homeostasis and promoting cell survival under long-term starvation. Our findings therefore provide insights into the molecular link between extracellular ROS and autophagy induction and suggest that MACP2-composed MAC pores at the plasma membrane may serve as functional channels for ROS influx in response to environmental or physiological stresses, including nutrient starvation.

## Materials and method

### Plant materials and growth conditions

Arabidopsis (*Arabidopsis thaliana*) accession Columbia-0 (Col-0) was used as the wild type in this study. The T-DNA insertion *macp2-1* (SALK_040186) and *macp2-2* (SALK_052845C) mutants were obtained from The Arabidopsis Information Resource (TAIR; http://www.arabidopsis.org). The *eds1-22*, *pad4*, *atg5-1*, *atg8e*, and *rpn10-1* mutants used in this study have been described previously (Shah 1997; Chen et al. 2015b; Xia et al. 2020). For genetic analysis, the *eds1-22* and *pad4* mutants were crossed to *MACP2-*OEs to generate the OE *eds1* (Zhang et al. 2022) and OE *pad4* lines; similarly, the *atg5-1* mutant was crossed to *macp2* mutants to generate the double mutants *macp2-1 atg5-1* and *macp2-2 atg5-1*. To investigate H_2_O_2_ influx in plants, *p35S::HyPer* transgenic line (Shi et al. 2022) was crossed to *macp2-1* mutant to generate the *macp2-1 p35S::HyPer* line. Fluorescence intensity o*f pHyPer* transgenic plants was determined in the roots of 1-week-old seedlings by the excitation wavelength at 488 nm or 405 nm and the emission at 530 nm (band pass 20). A fluorescence ratio was calculated as 488/405.

For seed germination assays, Arabidopsis seeds were surface-sterilized with 20% (v/v) bleach containing 0.1% (v/v) Tween 20 (Sigma, P2287) for 15 min, washed with distilled water at least six times, and plated on half-strength Murashige and Skoog (MS, Sigma, M5519) medium with 1% (w/v) sucrose and 0.8% (w/v) agar. The plates were incubated at 4 °C for 2 d in the darkness, before being transferred to a growth room under a 16-h-light/8-h-dark (20 °C/24 °C day/night) photoperiod. At 7 d after germination, seedlings were transplanted to soil (Pansa, PS008321) for further growth under the same conditions.

### Treatments

The treatment of nutrient starvation was performed as described previously (Qi et al. 2023). For nitrogen starvation tests, 7-d-old seedlings grown on solid half-strength MS medium were transferred to liquid half-strength MS medium or nitrogen-free liquid half-strength MS medium and grown under normal growth conditions for the indicated times. For carbon starvation, 4-week-old adult plants were transferred to continuous darkness for the indicated times and then allowed to recover under normal growth conditions for 7 d. For seedling treatment, 7-d-old seedlings grew in half-strength MS were transferred to solid sucrose-free medium with or without 500 μM GSH and incubated under continuous dark conditions for 12 d, followed by recovery under normal growth conditions for 5 d. Samples were collected or photographed at the indicated time points. The relative chlorophyll content was recorded as described below.

For chemical treatments, 7-d-old Arabidopsis seedlings grown on solid half-strength MS medium with 1% (w/v) sucrose were transferred to sterile 12-well plates filled with liquid half-strength MS medium with no added sucrose (− C) and 50 μM MG132 or 1 μM concanamycin A (ConA) in continuous darkness at the indicated time points (0, 6, 12, 24, or 48 h). After the specified treatments, seedlings were collected for confocal analysis or flash-frozen in liquid nitrogen for protein extraction and immunoblot analysis. For transient expression analysis, the plasmid *MACP2-HA* and *MACP2 M1-HA* were transfected into wild-type, *atg5-1*, *atg8e*, and *rpn10-1* protoplasts; after overnight culture under continuous light, protoplasts were treated with 50 μM cycloheximide (CHX) in darkness for the indicated times (0, 2, 4, or 6 h). Subsequently, the protoplasts were collected and the protein extracted for immunoblot analysis.

### Yeast Two-Hybrid (Y2H) screening

For conducting the yeast two-hybrid assay, the cDNA of *Arabidopsis ATG8e* was inserted into the pLexA vector, which was fused with the LexA DNA-binding domain (BD), thereby serving as the bait. The screening was carried out using the Matchmaker LexA two-hybrid system (Clontech) along with the EGY48 yeast strain to probe an Arabidopsis cDNA library (Xu et al. 2002). The transformed yeast cells were then cultured on 2% Gal/Raf/X-β-gal (SD-Ura/-His/-Trp/-Leu) selective medium in accordance with the guidelines provided by Clontech.

### Plasmid construction and transgenic plant generation

All constructs were generated using a ClonExpress II One Step Cloning Kit (catalog no. C112; Vazyme). All gene-specific primers with about 15-bp extensions homologous to the corresponding vectors are listed in Table S1. For the *MACP2-HA* and *MACP2 M1-HA* constructs, the full-length coding sequence of *MACP2* and *MACP2* with mutations (*MACP2*^*F134A*,*L137A*^) were cloned into *Bam*HI- and *Stu*I-digested pUC119 vector (containing the Arabidopsis *UBIQUITIN10* [*UBQ10*] promoter; Li et al. 2013; Xia et al. 2020) to generate the *MACP2-HA* and *MACP2 M1-HA* constructs. The full-length coding sequence ATG8e was cloned into *Bam*HI-digested pUC120 (harboring the Arabidopsis *UBQ10* promoter; Xia et al. 2020) to generate the *FLAG-eGFP-ATG8e* construct. To generate plasmids for the bimolecular fluorescence complementation (BiFC) assay, the full-length coding sequence of *MACP2* and its variant (*MACP2*^*F134A*, *L137A*^) was cloned into *Bam*HI-digested pHBT-YN or pHBT-YC vector (Qi et al. 2017), respectively, to generate *MACP2-nYFP* and M*ACP2 M1-nYFP* constructs. The full-length coding sequence fragment of *ATG8e* was cloned into *Bam*HI-digested pUC120-YC (Xia et al. 2020) to generate *cYFP-ATG8e* construct.

To generate stable transgenic plants expressing *MACP2-YFP* or *MACP2-FLAG*, the fusion cassettes derived from the above pUC119 constructs were cloned into the binary vector pFGC-RCS (Li et al. 2013; Zhang et al. 2022). The resulting clones were subsequently introduced into wild-type Arabidopsis (Col-0) or the mutants *eds1* and *pad4* via Agrobacterium (*Agrobacterium tumefaciens*) –mediated transformation using the floral dip method (Clough & Bent (Clough, et al., 1998)) to generate *MACP2-FLAG*, *MACP2-YFP* (*MACP2-*OEs), *MACP2-YFP eds1* (OE *eds1*), and *MACP2-YFP pad4* (OE *pad4*) transgenic plants. The *MACP2-YFP* line was crossed to *mCherry-ATG8f* to generate the double transgenic line *mCherry-ATG8f MACP2-YFP.* The *macp2-1 eGFP-ATG8e* double mutant was obtained by crossing *macp2-1* to the *eGFP-ATG8e* line, and the transgenic plant *MACP2-*OE *eGFP-ATG8e* (*MACP2-FLAG eGFP-ATG8e*) was produced by introduction of *MACP2-FLAG* into *eGFP-ATG8e* line.

### Co-immunoprecipitation (Co-IP) and BiFC assays

Preparation and transfection of Arabidopsis mesophyll protoplasts were performed according to Yoo et al. (2007). Protoplasts isolated from the rosettes of 4-week-old plants were transfected with the indicated plasmids and cultured for 16 h in the light before protein extraction. For the Co-IP assays, the transfected protoplasts were centrifuged and collected and lysed in 200 μL immunoprecipitation (IP) buffer (10 mM HEPES at pH 7.4, 150 mM NaCl, 2 mM EDTA, and 10% [v/v] glycerol) with 0.5% (v/v) Triton X-100. A 10% volume of total lysate was used as input, and the remainder was mixed with 300 μL IP buffer without Triton X-100 and incubated with FLAG affinity beads (Sigma-Aldrich) for 4 h at 4 °C. The beads were centrifuged, collected, and washed six times with IP buffer containing 0.1% (v/v) Triton X-100, followed by addition of SDS-PAGE loading buffer and heating at 95 °C for 5 min before immunoblot analysis.

For the BiFC assay, the appropriate pairs of *nYFP* and *cYFP* plasmids were transfected in leaf mesophyll protoplasts prepared from wild-type rosettes, incubated for 12 h under continuous light conditions to allow transgene expression, and then incubated with 100 μM benzo [1,2,3] thiadiazole-7-carbothioic acid S-methyl ester (BTH) and 1 μM ConA for 6 h for treated cells. The YFP fluorescence was detected by confocal microscopy (ZEISS 880) with an excitation wavelength of 514 nm and emission collected with a bandpass infrared (IR) filter from 524 to 550 nm.

### Measurement of chlorophyll content

Chlorophyll contents were determined as described previously (Qi et al. 2022). Arabidopsis leaves were harvested at the natural senescence stage or during carbon or nitrogen starvation at different growth periods. Total chlorophylls were extracted by incubating the samples in 2 mL anhydrous ethanol in the dark at 4 °C for 48 h. Absorbance was determined at 664 and 647 nm, and the total chlorophyll content was measured and normalized to fresh weight per sample.

### Measurement of phytohormone contents

Phytohormones were extracted and their concentrations measured as described previously (Pan et al. 2010; Chen et al. 2015a). Powdered tissue was weighed in a 2-mL centrifuge tube and extracted in 800 μL extraction buffer (2-propanol/water/concentrated HCl [2:1:0.005, v/v/v]) with internal standards (10 ng d4-salicylic acid [SA] and 10 ng d5-jasmonic acid [JA]; Sigma-Aldrich). The mixtures were mixed with shaking at 100 g for 30 min at 4 °C, after which 1 mL dichloromethane was added and shaking continued for an additional 30 min at 4 °C. The samples were then centrifuged at 13,000 g for 10 min at 4 °C. One milliliter from the lower phase was collected and dried using a nitrogen evaporator with nitrogen flow. The samples were dissolved in 200 μL 60% (v/v) methanol in sterile ultrapure water. Quantitative detection and analysis of phytohormones were performed with a chromatograph (Shimadzu) coupled to a mass spectrometer (Triple TOF 5600 [AB SCIEX]) (Chen et al. 2015a).

### Measurement of H_2_O_2_ content

H_2_O_2_ was extracted from the leaves of 4-, 5-, and 6-week-old Arabidopsis plants using an Amplex Red Hydrogen Peroxide/Peroxidase Assay Kit (Invitrogen) according to the manufacturer’s instructions, as described previously (Pucciariello et al. 2012). Briefly, 50 μL of extracted supernatant and 50 μL of the reaction working solution provided in the kit were transferred to a transparent plate and incubated at room temperature in darkness for 30 min. The absorption was determined at 560 nm on a multimode reader. A standard curve was generated by diluting the H_2_O_2_ working solution provided with the kit to concentrations of 2, 4, 6, 8, 10, 12, 15, or 20 μM in a final volume of 50 μL 1 × reaction buffer. Each dilution was mixed with 50 μL reaction working solution and incubated as above.

### RNA extraction and RT-qPCR

Total RNA was extracted from the leaves of 4-, 5-, and 6-week-old Arabidopsis plants using a HiPure Plant RNA Mini kit (Magen) according to the manufacturer’s instructions. The isolated total RNA was converted into first-strand cDNA with a HiScript II QRT Super Mix kit with gDNA Wiper (Vazyme). Quantitative PCR (qPCR) was performed with ChamQ SYBR Color qPCR Master Mix (Vazyme) on a StepOne Plus Real-time PCR System (Applied Biosystems).

qPCR was performed with the following settings: 95 °C for 5 min followed by 40 cycles of 95 °C for 15 s, 55 °C for 15 s, and 72 °C for 30 s, and a subsequent standard dissociation protocol to validate the presence of a unique polymerase chain reaction (PCR) product. For calculation of the relative expression levels of different genes, three technical replicates were performed per reaction. The delta of threshold cycle (△Ct) values were calculated by subtracting the arithmetic mean Ct values of the targets from the normalizing *ACTIN2*. The relative transcript level (2^−△△Ct^) was calculated from three independent experiments. The gene-specific primers used for qPCR are listed in Table S1.

### Protein extraction and immunoblot analysis

For total protein extraction, plant tissues were ground in liquid nitrogen and homogenized in ice-cold protein extraction buffer (50 mM sodium phosphate [pH 7.0], 200 mM NaCl, 10 mM MgCl_2_, 0.2% [v/v] β-mercaptoethanol, and 10% [v/v] glycerol) with protease inhibitor cocktail (Roche, 04693132001). The samples were placed on ice for 30 min and centrifuged at 4 °C for 10 min at 12,000 g. The supernatant was transferred to a new microfuge tube before electrophoresis. For total protein extraction from protoplasts, collected cells were flash-frozen in liquid nitrogen and extracted in IP buffer.

For immunoblot analysis, the supernatants from protein extracts were subjected to SDS-PAGE and transferred to a Hybond-C membrane (Amersham). Specific anti-HA (cat. no. H6533, 1:5,000; Sigma-Aldrich), anti-FLAG (cat. no. A8592, 1:5,000; Sigma-Aldrich), anti-eGFP (cat. no. M20004, 1:3,000; Abmart), and anti-ACTIN (cat. no. 58169; 1:5,000; Cell Signaling Technology) antibodies were used for immunoblotting analysis. Quantification of the immunoblot signal was determined with ImageJ software.

### Microscopy analyses

To determine the effect of *macp2* and *MACP2*-OE plants on autophagosome formation during carbon starvation, 7-d-old *eGFP-ATG8e*, *macp2-1 eGFP-ATG8e*, and *MACP2-*OE* eGFP-ATG8e* seedlings grown on half-strength MS medium were transferred to sucrose-free MS medium with or without 1 μM ConA and incubated in constant dark conditions for fixed-C starvation treatment. For H_2_O_2_ and BTH treatment, 7-d-old *eGFP-ATG8e*, *macp2-1 eGFP-ATG8e* and *MACP2-*OE* eGFP-ATG8e* seedlings were transferred to half-strength MS liquid medium with or without 100 μM H_2_O_2_ or BTH (SA agonist, benzo [1,2,3] thiadiazole-7-carbothioic acid S-methyl ester) for 12 h or 8 h, respectively.

For the subcellular localization of MACP2-YFP and the colocalization of MACP2-YFP with mCherry-ATG8f in response to nutrient starvation, 7-d-old *MACP2-YFP* and *MACP2-YFP mCherry-ATG8f* seedlings grown on solid half-strength MS medium were transferred to sucrose-free liquid half-strength MS medium (− C) containing 1 μM ConA and incubated in constant dark conditions for fixed-C starvation treatment, or seedlings were transferred to nitrogen-free liquid half-strength (− N) containing 1 μM ConA for indicated times. The eGFP, YFP and mCherry signals were detected by confocal microscopy (ZEISS 880) with an excitation wavelength of 488 nm (eGFP), 514 nm (YFP) or 561 nm (mCherry) and emission collected with a bandpass infrared (IR) filter from 500 to 530 nm (GFP, YFP), or 560 to 610 nm (mCherry).

### Statistical analysis

Data presented in this study are means ± standard deviation (SD) of three independent experiments unless indicated otherwise. Significant differences within or between groups were assessed by one-way analysis of variance (ANOVA) with Tukey’s honestly significant difference (HSD) test via STAT software, and a *P*-value < 0.05 was considered significant.

## Accession numbers

Sequence data from this article can be found in the Arabidopsis Genome Initiative database under the following accession numbers: *MACP1* (AT1G14780), *MACP2* (AT4G24290), *NSL1* (AT1G28380), *CAD1* (AT1G29690), *EXO70D1* (AT1G72470),

*EXO70D2* (AT1G54090), *EXO70D3* (AT3G14090), *ATG8e* (AT2G45170), *ATG8f* (AT4G16520), *ATG5* (AT5G17290), *EDS1* (AT3G48090), *PAD4* (AT3G52430), *SAG13* (AT2G29350), *SAG29* (AT5G13170), *SAG113* (AT5G59220), *SEN4* (AT4G30270), *PR1* (At2g14610), *PR5* (At1g75040), *ST1* (AT1G79230), *EDR2* (AT4G19040), and *WRKY53* (AT4G23810).

## Supplementary Information

Supplementary Material 1: Fig. S1.Structures of MACP proteins from Arabidopsis. Structures of NSL1 (AT1G28380), MACP1 (AT1G14780), and CAD1 (AT1G29690) were predicted by Alphafold and visualized using PyMOL software. AIM located within the proteins were analyzed by iLIR database. The predicted AIM is shown in red. Fig. S2. The accelerated senescence phenotypes of *MACP2-*OE plants are dependent on the salicylic acid pathway. a Representative photograph of Wild-type (WT), *MACP2-*OE-2,*eds1-22*,*pad4*, OE-2*eds1*, OE*-2pad4 seedlings* in response to nitrogen starvation (N−). For nitrogen starvation treatment, 7-d-old WT, OE-2,*eds1-22, pad4*, OE-2*eds1*, and OE-2*pad4*seedlings grown on half-strength solid MS medium were transferred to N-rich (N+) or N-free (N−) medium and photographed at 5 d into treatment. b Relative chlorophyll contents of 7-d-old wild-type (WT), *MACP2-*OE-1,*eds1-22*,*pad4*,OE-1*eds1-22*, and OE-1*pad4*plants seedlings under nitrogen deficiency, expressed as a percentage relative to control plants or seedlings. For each experiment,20 seedlings were used per genotype. All experiments were performed as three biological replicates, each with similar results. Relative chlorophyll contents are means ± SD (*n* = 3) calculated from three biological replicates. Different lowercase letters indicate significant differences within each group as determined by one-way ANOVA, *P* < 0.05. Fig. S3. The starvation sensitive phenotypes of *MACP2-*OE plants are rescued by GSH application. a Wild-type (WT), *MACP2-*OE (OE-1 and OE-2), and *atg5-1* seedlings grown on half-strength solid MS medium for 7 d. The seedlings were transferred to solid sucrose-rich medium (CK) or sucrose-free medium (C−) with or without 500 μM GSH and incubated under normal light/dark conditions or continuous dark conditions for 12 d, respectively, followed by recovery under normal growth conditions for 5 d. b Relative chlorophyll contents of 7-d-old WT, *MACP2-*OE (OE-1 and OE-2), and *atg5-1*plants in response to carbon starvation (a) for 12 d, followed by recovery under normal growth conditions for 5 d. For each experiment, 20 seedlings were used per genotype. All experiments were performed as three biological replicates, each with similar results. Relative chlorophyll contents are means ± SD (*n* = 3) calculated from three biological replicates. Different lowercase letters indicate significant differences between groups as determined by one-way ANOVA, *P* < 0.05. Fig. S4.Knockout of *MACP2* partially rescues the hypersensitive phenotypes of *atg5-1*to nitrogen deficiency. a and b Representative photographs (a) and relative chlorophyll contents (b) of wild-type (WT), *macp2-1*, *macp2-2*,*atg5-1*,*macp2-1 atg5-1*,and *macp2-2 atg5-1*double mutant seedlings in response to nitrogen starvation. 7-d-old WT*macp2*,*atg5-1*,*macp2-1 atg5-1*,and *macp2-2 atg5-1*double mutant seedlings grown on half-strength solid MS medium were transferred to N-rich (N+) or N-deficient (N−) medium and photographed at 5 d into treatment. For each experiment, 20 seedlings were used per genotype. The experiments were performed as three biological replicates, each with similar results. Data are means ± SD (*n* = 3) calculated from three biological replicates. Different lowercase letters indicate significant differences within each group as determined by one-way ANOVA, *P* < 0.05. Fig. S5. MACP2 modulates H_2_O_2_– or BTH–induced autophagosome formation. a and c Confocal analysis showing eGFP-ATG8e labeled autophagosomes in the root cells of wild-type (WT),*macp2-1*, and *MACP2*-OE seedlings. Seven-d-old*eGFP-ATG8e*, *macp2-1 eGFP-ATG8e*, and *MACP2-*OE*eGFP-ATG8e* (*MACP2-FLAG eGFP-ATG8e*) seedlings were transferred to half-strength MS liquid medium with or without 100 μM H_2_O_2_ or BTH (SA agonist, benzo [1,2,3] thiadiazole-7-carbothioic acid S-methyl ester) for 12 h or 8 h, respectively. The formation of autophagosomes was visualized by fluorescence confocal microscopy. Scale bars, 10 μm. banddNumber of puncta per root section in root cells from the *eGFP-ATG8e*, *macp2-1 eGFP-ATG8e*,and*MACP2*OE* eGFP-ATG8e*in (a and c). Data are means ± SD (*n*= 3) calculated from three biological replicates. For each experiment, 15 sections were used for calculation per genotype. Different lowercase letters indicate significant differences within each group as determined by one-way ANOVA, *P* < 0.05. Fig. S6. Colocalization of MACP2-YFP with mCherry-ATG8f in response to nitrogen starvation. A *MACP2-YFP* plant was crossed to *mCherry-ATG8f* to generate the double transgenic line *mCherry-ATG8f MACP2-YFP*. Colocalization assay of MACP2-YFP and mCherry-ATG8f in transgenic plants *mCherry-ATG8f MACP2-YFP*after 12 h of nitrogen starvation. YFP/mCherry was excited at 514/561 nm and the emissions were collected using a 554/601 nm band-pass emission filter on ZEISS LSM 880 confocal laser scanning microscope. Scale bars, 10 μm. Fig. S7. Identification of *MACP2-FLAG*transgenic plants. a and b Relative *MACP2*transcript levels (a) and accumulation of MACP2-FLAG(b) in 4-week-old wild-type (WT) and *pUBQ10:MACP2-FLAG*lines, as determined by RT-qPCR or immunoblot analysis, respectively. Anti-HA and anti-Actin antibodies were used for immunoblotting. Actin is shown as a loading control. The experiments were performed three times (biological replicates), each with similar results, and representative data from one replicate are shown. Data are means ± SD (*n* = 3) calculated from three technical replicates. Asterisks indicate significant differences from the WT (**P < 0.01, *P < 0.05 by Student’s *t* test). Fig. S8. Protein abundance of MACP2 and MACP2 M1 in wild type and *atg8e* mutant. The *GFP-FLAG* and *MACP2-HA* or *MACP2 M1-HA* plasmids were co-transfected into protoplasts isolated from wild type (WT) and *atg8e* mutant. After overnight culture, protoplasts were treated with 50 μM cycloheximide (CHX) under darkness for the indicated times (0, 4, 6, or 8 h). The experiments were performed as three biological replicates, each with similar results. The abundance of eGFP-FLAG was used as a control. The relative quantification of MACP2-HA and MACP2 MA-HA proteins were normalized to the corresponding eGFP-FLAG control and then compared to its level at 0 h. Data are means ± SD (*n* = 3) calculated from three biological replicates. The numbers on the left indicate the molecular weight (kD) of each size marker. hpt, h post-treatment.

Supplementary Material 2.

Supplementary Material 3.

## Data Availability

The original data and materials in the paper are available via contacting the corresponding author.

## References

[CR1] Acheampong AK, Shanks C, Cheng CY, Schaller GE, Dagdas Y, Kieber JJ. EXO70D isoforms mediate selective autophagic degradation of type-A ARR proteins to regulate cytokinin sensitivity. Proc Natl Acad Sci U S A. 2020;117:27034–43. 33051300 10.1073/pnas.2013161117 PMC7604425

[CR2] Asada Y, Yamamoto M, Tsutsui T, Yamaguchi J. The *Arabidopsis* NSL2 negatively controls systemic acquired resistance via hypersensitive response. Plant Biotechnol. 2011;28:9–15.

[CR3] Avila-Ospina L, Moison M, Yoshimoto K, Masclaux-Daubresse C. Autophagy, plant senescence, and nutrient recycling. J Exp Bot. 2014;65:3799–811. 24687977 10.1093/jxb/eru039

[CR4] Bakopoulos D, Whisstock JC, Johnson TK. Control of growth factor signalling by MACPF proteins. Biochem Soc Trans. 2019;47:801–10. 31209154 10.1042/BST20180179

[CR5] Bassham DC, Laporte M, Marty F, Moriyasu Y, Ohsumi Y, Olsen LJ, Yoshimoto K. Autophagy in development and stress responses of plants. Autophagy. 2006;2:2–11. 16874030 10.4161/auto.2092

[CR6] Castro B, Citterico M, Kimura S, Stevens DM, Wrzaczek M, Coaker G. Stress-induced reactive oxygen species compartmentalization, perception and signalling. Nat Plants. 2021;7:403–12. 33846592 10.1038/s41477-021-00887-0 PMC8751180

[CR7] Chen L, Liao B, Qi H, Xie LJ, Huang L, Tan WJ, Zhai N, Yuan LB, Zhou Y, Yu LJ, Chen QF, Shu WS, Xiao S. Autophagy contributes to regulation of the hypoxia response during submergence in *Arabidopsis thaliana*. Autophagy. 2015a;11:2233–46. 26566261 10.1080/15548627.2015.1112483 PMC4835207

[CR8] Chen QF, Xu L, Tan WJ, Chen L, Qi H, Xie LJ, Chen MX, Liu BY, Yu LJ, Yao N, Zhang JH, Shu W, Xiao S. Disruption of the Arabidopsis defense regulator genes SAG101, EDS1, and PAD4 confers enhanced freezing tolerance. Mol Plant. 2015b;8:1536–49. 26149542 10.1016/j.molp.2015.06.009 PMC5321072

[CR9] Chen P, Jian H, Wei F, Gu L, Hu T, Lv X, Guo X, Lu J, Ma L, Wang H, Wu A, Mao G, Yu S, Wei H. Phylogenetic analysis of the membrane attack complex/perforin domain-containing proteins in *Gossypium* and the role of *GhMACPF26* in cotton under cold stress. Front Plant Sci. 2021;12: 684227. 34868097 10.3389/fpls.2021.684227 PMC8641546

[CR10] Chung T, Phillips AR, Vierstra RD. ATG8 lipidation and ATG8-mediated autophagy in Arabidopsis require ATG12 expressed from the differentially controlled ATG12A and ATG12B loci. Plant J. 2010;62:483–93. 20136727 10.1111/j.1365-313X.2010.04166.x

[CR11] Clough SJ, Bent AF. Floral dip: a simplified method for *Agrobacterium*-mediated transformation of *Arabidopsis thaliana*. Plant J. 1998;16:735–43. 10069079 10.1046/j.1365-313x.1998.00343.x

[CR12] Essick EE, Flora S. Oxidative stress and autophagy in cardiac disease, neurological disorders, aging and cancer. Oxid Med Cell Longev. 2010;3:168–77. 20716941 10.4161/oxim.3.3.2 PMC2952075

[CR13] Fukunaga S, Sogame M, Hata M, Singkaravanit-Ogawa S, Pislewska-Bednarek M, Onozawa-Komori M, Nishiuchi T, Hiruma K, Saitoh H, Terauchi R, Kitakura S, Inoue Y, Bednarek P, Schulze-Lefert P, Takano Y. Dysfunction of *Arabidopsis* MACPF domain protein activates programmed cell death via tryptophan metabolism in MAMP-triggered immunity. Plant J. 2017;89:381–93. 27711985 10.1111/tpj.13391

[CR14] Grondin A, Rodrigues O, Verdoucq L, Merlot S, Leonhardt N, Maurel C. Aquaporins contribute to ABA-triggered stomatal closure through OST1-mediated phosphorylation. Plant Cell. 2015;27:1945–54. 26163575 10.1105/tpc.15.00421 PMC4531361

[CR15] Guan B, Lin Z, Liu D, Li C, Zhou Z, Mei F, Li J, Deng X. Effect of waterlogging-induced autophagy on programmed cell death in Arabidopsis roots. Front Plant Sci. 2019;10: 468. 31031792 10.3389/fpls.2019.00468 PMC6470631

[CR16] Guo Y, Ren G, Zhang K, Li Z, Miao Y, Guo H. Leaf senescence: progression, regulation, and application. Mol Hortic. 2021;1:5. 37789484 10.1186/s43897-021-00006-9 PMC10509828

[CR17] Halliwell B. Biochemistry of oxidative stress. Biochem Soc Trans. 2007;35:1147–50. 17956298 10.1042/BST0351147

[CR18] Hanaoka H, Noda T, Shirano Y, Kato T, Hayashi H, Shibata D, Tabata S, Ohsumi Y. Leaf senescence and starvation-induced chlorosis are accelerated by the disruption of an Arabidopsis autophagy gene. Plant Physiol. 2002;129:1181–93. 12114572 10.1104/pp.011024 PMC166512

[CR19] He Y, Gao J, Luo M, Gao C, Lin Y, Wong HY, Cui Y, Zhuang X, Jiang L. VAMP724 and VAMP726 are involved in autophagosome formation in Arabidopsis thaliana. Autophagy. 2023;13:19(5):1406–1423. 10.1080/15548627.2022.2127240 PMC10240985 36130166

[CR20] Hernández-Barrera A, Quinto C, Johnson EA, Wu HM, Cheung AY, Cárdenas L. Using hyper as a molecular probe to visualize hydrogen peroxide in living plant cells: a method with virtually unlimited potential in plant biology. Methods Enzymol. 2013;527:275–90. 23830637 10.1016/B978-0-12-405882-8.00015-5

[CR21] Holmes DR, Bredow M, Thor K, Pascetta SA, Sementchoukova I, Siegel KR, Zipfel C, Monaghan J. A novel allele of the Arabidopsis thaliana MACPF protein CAD1 results in deregulated immune signaling. Genetics. 2021;217:iyab022. 10.1093/genetics/iyab022 PMC8049551 33779749

[CR22] Huang L, Guo H. Acetylation modifcation in the regulation of macroautophagy. Adv Biotech. 2024;2: 19. 10.1007/s44307-024-00027-7 PMC11740868 39883319

[CR23] Huang L, Yu LJ, Zhang X, Fan B, Wang FZ, Dai YS, Qi H, Zhou Y, Xie LJ, Xiao S. Autophagy regulates glucose-mediated root meristem activity by modulating ROS production in Arabidopsis. Autophagy. 2019;15:407–22. 30208757 10.1080/15548627.2018.1520547 PMC6351127

[CR24] Kaufmann A, Wollert T. Scaffolding the expansion of autophagosomes. Autophagy. 2014;10:1343–5. 24963637 10.4161/auto.28980 PMC4203560

[CR25] Kaufmann A, Beier V, Franquelim HG, Wollert T. Molecular mechanism of autophagic membrane-scaffold assembly and disassembly. Cell. 2014;156:469–81. 24485455 10.1016/j.cell.2013.12.022

[CR26] Kellner R, de la Concepcion JC, Maqbool A, Kamoun S, Dagdas YF. ATG8 expansion: a driver of selective autophagy diversification? Trends Plant Sci. 2017;22:204–14. 28038982 10.1016/j.tplants.2016.11.015

[CR27] Krüger C, Waldeck-Weiermair M, Kaynert J, Pokrant T, Komaragiri Y, Otto O, Michel T, Elsner M. AQP8 is a crucial H_2_O_2_ transporter in insulin-producing RINm5F cells. Redox Biol. 2021;43: 101962. 33892285 10.1016/j.redox.2021.101962 PMC8082690

[CR28] Li F, Vierstra RD. Autophagy: a multifaceted intracellular system for bulk and selective recycling. Trends Plant Sci. 2012;17:526–37. 22694835 10.1016/j.tplants.2012.05.006

[CR29] Li JF, Chung HS, Niu Y, Bush J, Mc Cormack M, Sheen J. Comprehensive protein-based artificial microRNA screens for effective gene silencing in plants. Plant Cell. 2013;25:1507–22. 23645631 10.1105/tpc.113.112235 PMC3694689

[CR30] Li X, Liu Q, Feng H, Deng J, Zhang R, Wen J, Dong J, Wang T. Dehydrin MtCAS31 promotes autophagic degradation under drought stress. Autophagy. 2020;16:862–77. 31362589 10.1080/15548627.2019.1643656 PMC7144882

[CR31] Liao CY, Bassham DC. Combating stress: the interplay between hormone signaling and autophagy in plants. J Exp Bot. 2020;71:1723–33. 31725881 10.1093/jxb/erz515 PMC7067298

[CR32] Lin Z, Wang YL, Cheng LS, Zhou LL, Xu QT, Liu DC, Deng XY, Mei FZ, Zhou ZQ. Mutual regulation of ROS accumulation and cell autophagy in wheat roots under hypoxia stress. Plant Physiol Biochem. 2021;158:91–102. 33302125 10.1016/j.plaphy.2020.11.049

[CR33] Liu Y, Bassham DC. Autophagy: pathways for self-eating in plant cells. Annu Rev Plant Biol. 2012;63:215–37. 22242963 10.1146/annurev-arplant-042811-105441

[CR34] Lukoyanova N, Hoogenboom BW, Saibil HR. The membrane attack complex, perforin and cholesterol-dependent cytolysin superfamily of pore-forming proteins. J Cell Sci. 2016;129:2125–33. 27179071 10.1242/jcs.182741

[CR35] Marshall RS, Li F, Gemperline DC, Book AJ, Vierstra RD. Autophagic degradation of the 26S proteasome is mediated by the dual ATG8/ubiquitin receptor RPN10 in Arabidopsis. Mol Cell. 2015;58:1053–66. 26004230 10.1016/j.molcel.2015.04.023 PMC4903074

[CR36] Marshall RS, Hua Z, Mali S, McLoughlin F, Vierstra RD. ATG8-binding UIM proteins define a new class of autophagy adaptors and receptors. Cell. 2019;177:766–81. 30955882 10.1016/j.cell.2019.02.009 PMC6810650

[CR37] Mittler R, Zandalinas SI, Fichman Y, Van Breusegem F. Reactive oxygen species signalling in plant stress responses. Nat Rev Mol Cell Biol. 2022;23:663–79. 35760900 10.1038/s41580-022-00499-2

[CR38] Moreno-Hagelsieb G, Vitug B, Medrano-Soto A, Saier MH Jr. The membrane attack complex/perforin superfamily. J Mol Microb Biotech. 2017;27:252–67. 10.1159/000481286 PMC5796659 29145176

[CR39] Morgan BP. Complement membrane attack on nucleated cells: resistance, recovery and non-lethal effects. Biochem J. 1989;264:1–14. 2690818 10.1042/bj2640001 PMC1133540

[CR40] Morita-Yamamuro C, Tsutsui T, Sato M, Yoshioka H, Tamaoki M, Ogawa D, Matsuura H, Yoshihara T, Ikeda A, Uyeda I, Yamaguchi J. The Arabidopsis gene CAD1 controls programmed cell death in the plant immune system and encodes a protein containing a MACPF domain. Plant Cell Physiol. 2005;46:902–12. 15799997 10.1093/pcp/pci095

[CR41] Nakatogawa H, Ohbayashi S, Sakoh-Nakatogawa M, Kakuta S, Suzuki SW, Kirisako H, Kondo-Kakuta C, Noda NN, Yamamoto H, Ohsumi Y. The autophagy-related protein kinase Atg1 interacts with the ubiquitin-like protein Atg8 via the Atg8 family interacting motif to facilitate autophagosome formation. J Biol Chem. 2012;287:28503–7. 22778255 10.1074/jbc.C112.387514 PMC3436563

[CR42] Nazio F, Carinci M, Valacca C, Bielli P, Strappazzon F, Antonioli M, Ciccosanti F, Rodolfo C, Campello S, Fimia GM, Sette C, Bonaldo P, Cecconi F. Fine-tuning of ULK1 mRNA and protein levels is required for autophagy oscillation. J Cell Biol. 2016;215:841–56. 27932573 10.1083/jcb.201605089 PMC5166502

[CR43] Nazio F, Carinci M, Cecconi F. ULK1 ubiquitylation is regulated by phosphorylation on its carboxy terminus. Cell Cycle. 2017;16:1744–7. 28820317 10.1080/15384101.2017.1361063 PMC5628632

[CR44] Ni T, Gilbert RJC. Repurposing a pore: highly conserved perforin-like proteins with alternative mechanisms. Philos Trans R Soc Lond B Biol Sci. 2017;372: 20160212. 28630152 10.1098/rstb.2016.0212 PMC5483515

[CR45] Noutoshi Y, Kuromori T, Wada T, Hirayama T, Kamiya A, Imura Y, Yasuda M, Nakashita H, Shirasu K, Shinozaki K. Loss of necrotic spotted lesions 1 associates with cell death and defense responses in *Arabidopsis thaliana*. Plant Mol Biol. 2006;62:29–42. 16900325 10.1007/s11103-006-9001-6

[CR46] Pan X, Welti R, Wang X. Quantitative analysis of major plant hormones in crude plant extracts by high-performance liquid chromatography-mass spectrometry. Nat Protoc. 2010;5:986–92. 20448544 10.1038/nprot.2010.37

[CR47] Pucciariello C, Parlanti S, Banti V, Novi G, Perata P. Reactive oxygen species-driven transcription in Arabidopsis under oxygen deprivation. Plant Physiol. 2012;159:184–96. 22415514 10.1104/pp.111.191122 PMC3375960

[CR48] Qi H, Xia FN, Xie LJ, Yu LJ, Chen QF, Zhuang XH, Wang Q, Li F, Jiang L, Xie Q, Xiao S. TRAF family proteins regulate autophagy dynamics by modulating AUTOPHAGY PROTEIN6 stability in Arabidopsis. Plant Cell. 2017;29:890–911. 28351989 10.1105/tpc.17.00056 PMC5435438

[CR49] Qi H, Xia FN, Xiao S. Autophagy in plants: Physiological roles and post-translational regulation. J Integr Plant Biol. 2021;63:161–79. 32324339 10.1111/jipb.12941

[CR50] Qi H, Lei X, Wang Y, Yu S, Liu T, Zhou SK, Chen JY, Chen QF, Qiu RL, Jiang L, Xiao S. 14-3-3 proteins contribute to autophagy by modulating SINAT-mediated degradation of ATG13. Plant Cell. 2022;34:4857–76. 36053201 10.1093/plcell/koac273 PMC9709989

[CR51] Qi H, Wang Y, Bao Y, Bassham DC, Chen L, Chen QF, Hou SW, Hwang I, Huang L, Lai ZB, Li F, Liu Y, Qiu R, Wang H, Wang P, Xie Q, Zeng Y, Zhuang X, Gao C, Jiang L, Xiao S. Studying plant autophagy: challenges and recommended methodologies. Adv Biotechnol. 2023;1:2. 10.1007/s44307-023-00002-8 PMC11727600 39883189

[CR52] Qu X, Chatty PR, Roeder AHK. Endomembrane trafficking protein SEC24A regulates cell size patterning in Arabidopsis. Plant Physiol. 2014;166:1877–90. 25315606 10.1104/pp.114.246033 PMC4256882

[CR53] Rosado CJ, Kondos S, Bull TE, Kuiper MJ, Law RH, Buckle AM, Voskoboinik I, Bird PI, Trapani JA, Whisstock JC, Dunstone MA. The MACPF/CDC family of pore-forming toxins. Cell Microbiol. 2008;10:1765–74. 18564372 10.1111/j.1462-5822.2008.01191.x PMC2654483

[CR54] Scherz-Shouval R, Shvets E, Fass E, Shorer H, Gil L, Elazar Z. Reactive oxygen species are essential for autophagy and specifically regulate the activity of Atg4. EMBO J. 2007;26:1749–60. 17347651 10.1038/sj.emboj.7601623 PMC1847657

[CR55] Shah J. The salicylic acid loop in plant defense. Curr Opin Plant Biol. 2003;6:365–71. 12873532 10.1016/s1369-5266(03)00058-x

[CR56] Shah J, Tsui F, Klessig DF. Characterization of a salicylic acid-insensitive mutant (*sai1*) of *Arabidopsis thaliana*, identified in a selective screen utilizing the SA-inducible expression of the *tms2* gene. Mol Plant Microbe Interact. 1997;10:69–78. 9002272 10.1094/MPMI.1997.10.1.69

[CR57] Sharma P, Jha AB, Dubey RS, Pessarakli M. Reactive oxygen species, oxidative damage, and antioxidative defense mechanism in plants under stressful conditions. J Bot. 2012;2012:217037. 10.1155/2012/217037.

[CR58] Shi W, Wang L, Yao L, Hao W, Han C, Fan M, Wang W, Bai MY. Spatially patterned hydrogen peroxide orchestrates stomatal development in Arabidopsis. Nat Commun. 2022;13(1): 5040. 36028510 10.1038/s41467-022-32770-7 PMC9418256

[CR59] Shin JH, Yoshimoto K, Ohsumi Y, Jeon JS, An G. OsATG10b, an autophagosome component, is needed for cell survival against oxidative stresses in rice. Mol Cell. 2009;27:67–74. 10.1007/s10059-009-0006-2 19214435

[CR60] Signorelli S, Tarkowski ŁP, Van den Ende W, Bassham DC. Linking autophagy to abiotic and biotic stress responses. Trends Plant Sci. 2019;24:413–30. 30824355 10.1016/j.tplants.2019.02.001 PMC6475611

[CR61] Smalle J, Kurepa J, Yang P, Emborg TJ, Babiychuk E, Kushnir S, Vierstra RD. The pleiotropic role of the 26S proteasome subunit RPN10 in Arabidopsis growth and development supports a substrate-s pecific function in abscisic acid signaling. Plant Cell. 2003;2003(15):965–80. 10.1105/tpc.009217 PMC152342 12671091

[CR62] Suttangkakul A, Li F, Chung T, Vierstra RD. The ATG1/ATG13 protein kinase complex is both a regulator and a target of autophagic recycling in Arabidopsis. Plant Cell. 2011;23(10):3761–79. 21984698 10.1105/tpc.111.090993 PMC3229148

[CR63] Tang J, Bassham DC. Autophagy during drought: function, regulation, and potential application. Plant J. 2022;109:390–401. 34469611 10.1111/tpj.15481

[CR64] Tian S, Wang X, Li P, Wang H, Ji H, Xie J, Qiu Q, Shen D, Dong H. Plant aquaporin AtPIP1;4 links apoplastic H_2_O_2_ induction to disease immunity pathways. Plant Physiol. 2016;171(3):1635–50. 26945050 10.1104/pp.15.01237 PMC4936539

[CR65] Tsutsui T, Morita-Yamamuro C, Asada Y, Minami E, Shibuya N, Ikeda A, Yamaguchi J. Salicylic acid and a chitin elicitor both control expression of the CAD1 gene involved in the plant immunity of Arabidopsis. Biosci Biotechnol Biochem. 2006;70:2042–8. 16960394 10.1271/bbb.50700

[CR66] Valkai I, Kénesi F, Domonkos I, Ayaydin F, Tarkowská D, Strnad M, Faragó A, Bodai L, Fehér A. The Arabidopsis RLCK VI_A2 kinase controls seedling and plant growth in parallel with gibberellin. Int J Mol Sci. 2020;21: 7266. 33019674 10.3390/ijms21197266 PMC7582978

[CR67] Wada S, Ishida H, Izumi M, Yoshimoto K, Ohsumi Y, Mae T, Makino A. Autophagy plays a role in chloroplast degradation during senescence in individually darkened leaves. Plant Physiol. 2009;149:885–93. 19074627 10.1104/pp.108.130013 PMC2633819

[CR68] Waszczak C, Carmody M, Kangasjarvi J. Reactive oxygen species in plant signaling. Annu Rev Plant Biol. 2018;69:209–36. 29489394 10.1146/annurev-arplant-042817-040322

[CR69] Weindel CG, Martinez EL, Zhao X, Mabry CJ, Bell SL, Vail KJ, Coleman AK, VanPortfliet JJ, Zhao B, Wagner AR, Azam S, Scott HM, Li P, West AP, Karpac J, Patrick KL, Watson RO. Mitochondrial ROS promotes susceptibility to infection via gasdermin D-mediated necroptosis. Cell. 2022;185:3214–31. 35907404 10.1016/j.cell.2022.06.038 PMC9531054

[CR70] Xia FN, Zeng B, Liu HS, Qi H, Xie LJ, Yu LJ, Chen QF, Li JF, Chen YQ, Jiang L, Xiao S. SINAT E3 ubiquitin ligases mediate FREE1 and VPS23A degradation to modulate abscisic acid signaling. Plant Cell. 2020;32:3290–310. 32753431 10.1105/tpc.20.00267 PMC7534459

[CR71] Xiao S, Gao W, Chen QF, Chan SW, Zheng SX, Ma J, Wang M, Welti R, Chye ML. Overexpression of Arabidopsis Acyl-CoA binding protein ACBP3 promotes starvation-induced and age-dependent leaf senescence. Plant Cell. 2010;22:1463–82. 20442372 10.1105/tpc.110.075333 PMC2899868

[CR72] Xiong Y, Contento AL, Nguyen PQ, Bassham DC. Degradation of oxidized proteins by autophagy during oxidative stress in Arabidopsis. Plant Physiol. 2007;143:291–9. 17098847 10.1104/pp.106.092106 PMC1761971

[CR73] Xu L, Liu F, Lechner E, Genschik P, Crosby WL, Ma H, Peng W, Huang D, Xie D. The SCF (COI1) ubiquitin-ligase complexes are required for jasmonate response in Arabidopsis. Plant Cell. 2002;14:1919–35. 12172031 10.1105/tpc.003368 PMC151474

[CR74] Yoo SD, Cho YH, Sheen J. *Arabidopsis* mesophyll protoplasts: a versatile cell system for transient gene expression analysis. Nat Protoc. 2007;2:1565–72. 17585298 10.1038/nprot.2007.199

[CR75] Yoshimoto K, Hanaoka H, Sato S, Kato T, Tabata S, Noda T, Ohsumi Y. Processing of ATG8s, ubiquitin-like proteins, and their deconjugation by ATG4s are essential for plant autophagy. Plant Cell. 2004;16:2967–83. 15494556 10.1105/tpc.104.025395 PMC527192

[CR76] Yoshimoto K, Jikumaru Y, Kamiya Y, Kusano M, Consonni C, Panstruga R, Ohsumi Y, Shirasu K. Autophagy negatively regulates cell death by controlling NPR1-dependent salicylic acid signaling during senescence and the innate immune response in Arabidopsis. Plant Cell. 2009;21:2914–27. 19773385 10.1105/tpc.109.068635 PMC2768913

[CR77] Yu L, Liu D, Chen S, Dai Y, Guo W, Zhang X, Wang L, Ma S, Xiao M, Qi H, Xiao S, Chen QF. Evolution and expression of the membrane attack complex and perforin gene family in the Poaceae. Int J Mol Sci. 2020;21: 5736. 32785137 10.3390/ijms21165736 PMC7460961

[CR78] Yuan LB, Dai YS, Xie LJ, Yu LJ, Zhou Y, Lai YX, Yang YC, Xu L, Chen QF, Xiao S. Jasmonate regulates plant responses to postsubmergence reoxygenation through transcriptional activation of antioxidant synthesis. Plant Physiol. 2017;173:1864–80. 28082717 10.1104/pp.16.01803 PMC5338657

[CR79] Zhang X, Dai YS, Wang YX, Su ZZ, Yu LJ, Zhang ZF, Xiao S, Chen QF. Overexpression of the Arabidopsis MACPF protein AtMACP2 promotes pathogen resistance by activating SA signaling. Int J Mol Sci. 2022;23:8784. 10.3390/ijms23158784. 10.3390/ijms23158784 PMC9369274 35955922

[CR80] Zhou J, Li XY, Liu YJ, Feng J, Wu Y, Shen HM, Lu GD. Full-coverage regulations of autophagy by ROS: from induction to maturation. Autophagy. 2022;18:1240–55. 34662529 10.1080/15548627.2021.1984656 PMC9225210

[CR81] Zhuang X, Chung KP, Luo M, Jiang L. Autophagosome biogenesis and the endoplasmic reticulum: a plant perspective. Trends Plant Sci. 2018;23:677–92. 29929776 10.1016/j.tplants.2018.05.002

[CR82] Zou JJ, Li XD, Ratnasekera D, Wang C, Liu WX, Song LF, Zhang WZ, Wu WH. *Arabidopsis* CALCIUM-DEPENDENT PROTEIN KINASE8 and CATALASE3 function in abscisic acid-mediated signaling and H_2_O_2_ homeostasis in stomatal guard cells under drought stress. Plant Cell. 2015;27:1445–60. 25966761 10.1105/tpc.15.00144 PMC4456645

