# Supplementary figures and images for: Arabidopsis MACP2 contributes to autophagy induction by modulating starvation-induced reactive oxygen species homeostasis
Source: Adv Biotechnol (Singap). 2025 Aug 20;3(3):25. doi: 10.1007/s44307-025-00078-4 (PMC12367599; doi:10.1007/s44307-025-00078-4)

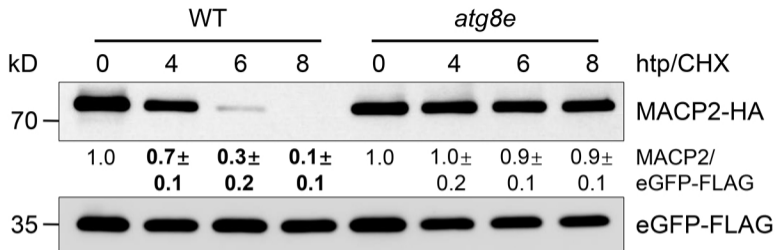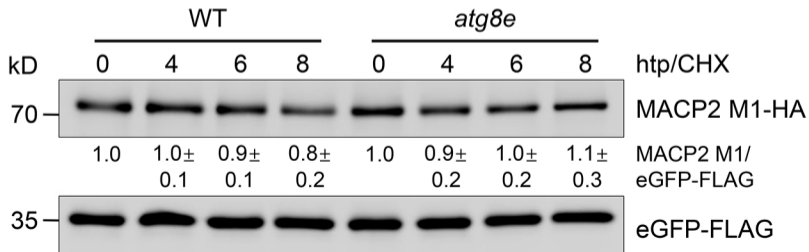

Supplement: Supplementary file 1 — Supplementary Material 1: Fig. S1.Structures of MACP proteins from Arabidopsis. Structures of NSL1 (AT1G28380), MACP1 (AT1G14780), and CAD1 (AT1G29690) were predicted by Alphafold and visualized using PyMOL software. AIM located within the proteins were analyzed by iLIR database. The predicted AIM is shown in red. Fig. S2. The accelerated senescence phenotypes of MACP2-OE plants are dependent on the salicylic acid pathway. a Representative photograph of Wild-type (WT), MACP2-OE-2,eds1-22,pad4, OE-2eds1, OE-2pad4 seedlings in response to nitrogen starvation (N−). For nitrogen starvation treatment, 7-d-old WT, OE-2,eds1-22, pad4, OE-2eds1, and OE-2pad4seedlings grown on half-strength solid MS medium were transferred to N-rich (N+) or N-free (N−) medium and photographed at 5 d into treatment. b Relative chlorophyll contents of 7-d-old wild-type (WT), MACP2-OE-1,eds1-22,pad4,OE-1eds1-22, and OE-1pad4plants seedlings under nitrogen deficiency, expressed as a percentage relative to control plants or seedlings. For each experiment,20 seedlings were used per genotype. All experiments were performed as three biological replicates, each with similar results. Relative chlorophyll contents are means ± SD (n = 3) calculated from three biological replicates. Different lowercase letters indicate significant differences within each group as determined by one-way ANOVA, P < 0.05. Fig. S3. The starvation sensitive phenotypes of MACP2-OE plants are rescued by GSH application. a Wild-type (WT), MACP2-OE (OE-1 and OE-2), and atg5-1 seedlings grown on half-strength solid MS medium for 7 d. The seedlings were transferred to solid sucrose-rich medium (CK) or sucrose-free medium (C−) with or without 500 μM GSH and incubated under normal light/dark conditions or continuous dark conditions for 12 d, respectively, followed by recovery under normal growth conditions for 5 d. b Relative chlorophyll contents of 7-d-old WT, MACP2-OE (OE-1 and OE-2), and atg5-1plants in response to carbon starvatio [file 44307_2025_78_MOESM1_ESM.zip › S8_ESM.pdf]

**a**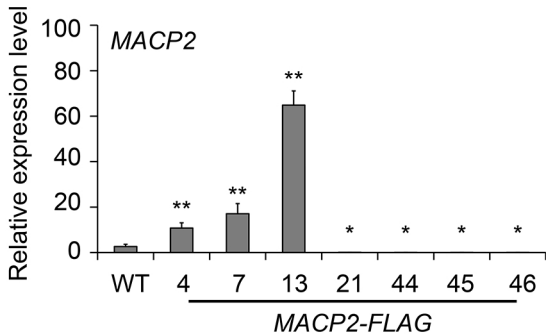**b**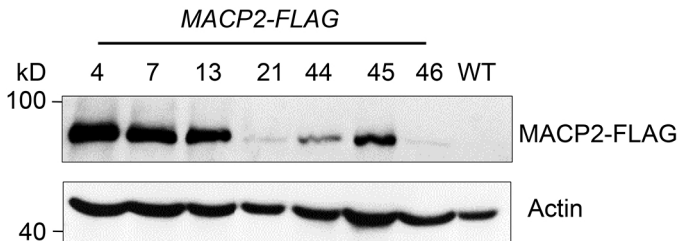

Supplement: Supplementary file 1 — Supplementary Material 1: Fig. S1.Structures of MACP proteins from Arabidopsis. Structures of NSL1 (AT1G28380), MACP1 (AT1G14780), and CAD1 (AT1G29690) were predicted by Alphafold and visualized using PyMOL software. AIM located within the proteins were analyzed by iLIR database. The predicted AIM is shown in red. Fig. S2. The accelerated senescence phenotypes of MACP2-OE plants are dependent on the salicylic acid pathway. a Representative photograph of Wild-type (WT), MACP2-OE-2,eds1-22,pad4, OE-2eds1, OE-2pad4 seedlings in response to nitrogen starvation (N−). For nitrogen starvation treatment, 7-d-old WT, OE-2,eds1-22, pad4, OE-2eds1, and OE-2pad4seedlings grown on half-strength solid MS medium were transferred to N-rich (N+) or N-free (N−) medium and photographed at 5 d into treatment. b Relative chlorophyll contents of 7-d-old wild-type (WT), MACP2-OE-1,eds1-22,pad4,OE-1eds1-22, and OE-1pad4plants seedlings under nitrogen deficiency, expressed as a percentage relative to control plants or seedlings. For each experiment,20 seedlings were used per genotype. All experiments were performed as three biological replicates, each with similar results. Relative chlorophyll contents are means ± SD (n = 3) calculated from three biological replicates. Different lowercase letters indicate significant differences within each group as determined by one-way ANOVA, P < 0.05. Fig. S3. The starvation sensitive phenotypes of MACP2-OE plants are rescued by GSH application. a Wild-type (WT), MACP2-OE (OE-1 and OE-2), and atg5-1 seedlings grown on half-strength solid MS medium for 7 d. The seedlings were transferred to solid sucrose-rich medium (CK) or sucrose-free medium (C−) with or without 500 μM GSH and incubated under normal light/dark conditions or continuous dark conditions for 12 d, respectively, followed by recovery under normal growth conditions for 5 d. b Relative chlorophyll contents of 7-d-old WT, MACP2-OE (OE-1 and OE-2), and atg5-1plants in response to carbon starvatio [file 44307_2025_78_MOESM1_ESM.zip › S7_ESM.pdf]

MACP2-YFP

N- + ConA

mCherry-ATG8f

Overlay

5 X Enlarged

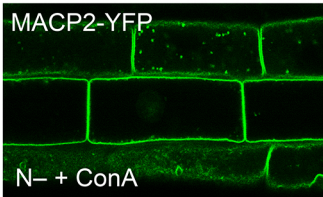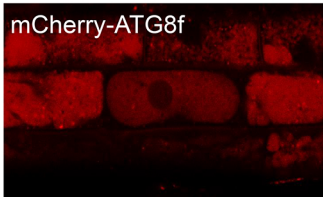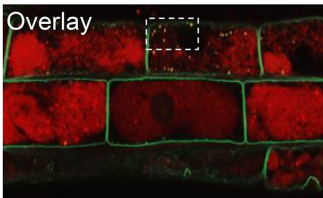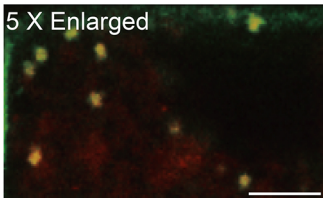

Supplement: Supplementary file 1 — Supplementary Material 1: Fig. S1.Structures of MACP proteins from Arabidopsis. Structures of NSL1 (AT1G28380), MACP1 (AT1G14780), and CAD1 (AT1G29690) were predicted by Alphafold and visualized using PyMOL software. AIM located within the proteins were analyzed by iLIR database. The predicted AIM is shown in red. Fig. S2. The accelerated senescence phenotypes of MACP2-OE plants are dependent on the salicylic acid pathway. a Representative photograph of Wild-type (WT), MACP2-OE-2,eds1-22,pad4, OE-2eds1, OE-2pad4 seedlings in response to nitrogen starvation (N−). For nitrogen starvation treatment, 7-d-old WT, OE-2,eds1-22, pad4, OE-2eds1, and OE-2pad4seedlings grown on half-strength solid MS medium were transferred to N-rich (N+) or N-free (N−) medium and photographed at 5 d into treatment. b Relative chlorophyll contents of 7-d-old wild-type (WT), MACP2-OE-1,eds1-22,pad4,OE-1eds1-22, and OE-1pad4plants seedlings under nitrogen deficiency, expressed as a percentage relative to control plants or seedlings. For each experiment,20 seedlings were used per genotype. All experiments were performed as three biological replicates, each with similar results. Relative chlorophyll contents are means ± SD (n = 3) calculated from three biological replicates. Different lowercase letters indicate significant differences within each group as determined by one-way ANOVA, P < 0.05. Fig. S3. The starvation sensitive phenotypes of MACP2-OE plants are rescued by GSH application. a Wild-type (WT), MACP2-OE (OE-1 and OE-2), and atg5-1 seedlings grown on half-strength solid MS medium for 7 d. The seedlings were transferred to solid sucrose-rich medium (CK) or sucrose-free medium (C−) with or without 500 μM GSH and incubated under normal light/dark conditions or continuous dark conditions for 12 d, respectively, followed by recovery under normal growth conditions for 5 d. b Relative chlorophyll contents of 7-d-old WT, MACP2-OE (OE-1 and OE-2), and atg5-1plants in response to carbon starvatio [file 44307_2025_78_MOESM1_ESM.zip › S6_ESM.pdf]

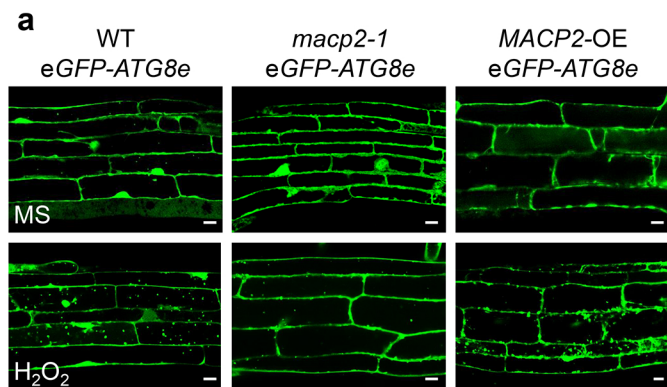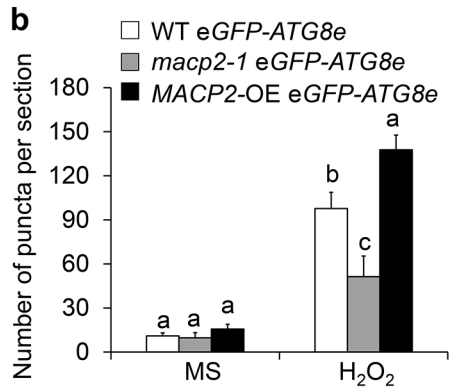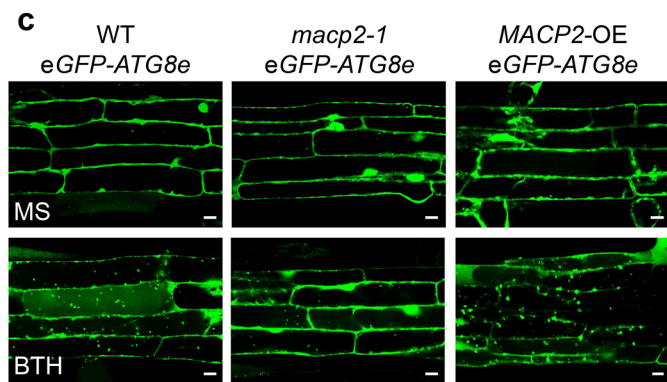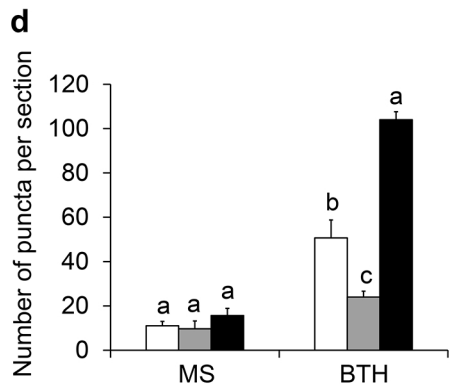

Supplement: Supplementary file 1 — Supplementary Material 1: Fig. S1.Structures of MACP proteins from Arabidopsis. Structures of NSL1 (AT1G28380), MACP1 (AT1G14780), and CAD1 (AT1G29690) were predicted by Alphafold and visualized using PyMOL software. AIM located within the proteins were analyzed by iLIR database. The predicted AIM is shown in red. Fig. S2. The accelerated senescence phenotypes of MACP2-OE plants are dependent on the salicylic acid pathway. a Representative photograph of Wild-type (WT), MACP2-OE-2,eds1-22,pad4, OE-2eds1, OE-2pad4 seedlings in response to nitrogen starvation (N−). For nitrogen starvation treatment, 7-d-old WT, OE-2,eds1-22, pad4, OE-2eds1, and OE-2pad4seedlings grown on half-strength solid MS medium were transferred to N-rich (N+) or N-free (N−) medium and photographed at 5 d into treatment. b Relative chlorophyll contents of 7-d-old wild-type (WT), MACP2-OE-1,eds1-22,pad4,OE-1eds1-22, and OE-1pad4plants seedlings under nitrogen deficiency, expressed as a percentage relative to control plants or seedlings. For each experiment,20 seedlings were used per genotype. All experiments were performed as three biological replicates, each with similar results. Relative chlorophyll contents are means ± SD (n = 3) calculated from three biological replicates. Different lowercase letters indicate significant differences within each group as determined by one-way ANOVA, P < 0.05. Fig. S3. The starvation sensitive phenotypes of MACP2-OE plants are rescued by GSH application. a Wild-type (WT), MACP2-OE (OE-1 and OE-2), and atg5-1 seedlings grown on half-strength solid MS medium for 7 d. The seedlings were transferred to solid sucrose-rich medium (CK) or sucrose-free medium (C−) with or without 500 μM GSH and incubated under normal light/dark conditions or continuous dark conditions for 12 d, respectively, followed by recovery under normal growth conditions for 5 d. b Relative chlorophyll contents of 7-d-old WT, MACP2-OE (OE-1 and OE-2), and atg5-1plants in response to carbon starvatio [file 44307_2025_78_MOESM1_ESM.zip › S5_ESM.pdf]

**a**

WT    *macp2-1*    *macp2-2*    *macp2-1 atg5-1*    *macp2-2 atg5-1*    *atg5-1*

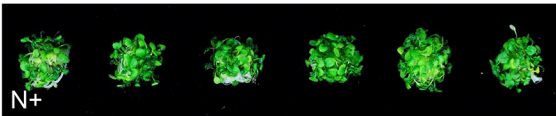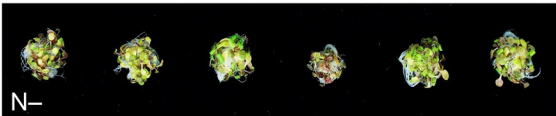**b**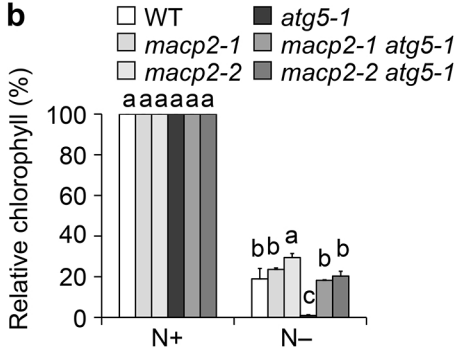

Supplement: Supplementary file 1 — Supplementary Material 1: Fig. S1.Structures of MACP proteins from Arabidopsis. Structures of NSL1 (AT1G28380), MACP1 (AT1G14780), and CAD1 (AT1G29690) were predicted by Alphafold and visualized using PyMOL software. AIM located within the proteins were analyzed by iLIR database. The predicted AIM is shown in red. Fig. S2. The accelerated senescence phenotypes of MACP2-OE plants are dependent on the salicylic acid pathway. a Representative photograph of Wild-type (WT), MACP2-OE-2,eds1-22,pad4, OE-2eds1, OE-2pad4 seedlings in response to nitrogen starvation (N−). For nitrogen starvation treatment, 7-d-old WT, OE-2,eds1-22, pad4, OE-2eds1, and OE-2pad4seedlings grown on half-strength solid MS medium were transferred to N-rich (N+) or N-free (N−) medium and photographed at 5 d into treatment. b Relative chlorophyll contents of 7-d-old wild-type (WT), MACP2-OE-1,eds1-22,pad4,OE-1eds1-22, and OE-1pad4plants seedlings under nitrogen deficiency, expressed as a percentage relative to control plants or seedlings. For each experiment,20 seedlings were used per genotype. All experiments were performed as three biological replicates, each with similar results. Relative chlorophyll contents are means ± SD (n = 3) calculated from three biological replicates. Different lowercase letters indicate significant differences within each group as determined by one-way ANOVA, P < 0.05. Fig. S3. The starvation sensitive phenotypes of MACP2-OE plants are rescued by GSH application. a Wild-type (WT), MACP2-OE (OE-1 and OE-2), and atg5-1 seedlings grown on half-strength solid MS medium for 7 d. The seedlings were transferred to solid sucrose-rich medium (CK) or sucrose-free medium (C−) with or without 500 μM GSH and incubated under normal light/dark conditions or continuous dark conditions for 12 d, respectively, followed by recovery under normal growth conditions for 5 d. b Relative chlorophyll contents of 7-d-old WT, MACP2-OE (OE-1 and OE-2), and atg5-1plants in response to carbon starvatio [file 44307_2025_78_MOESM1_ESM.zip › S4_ESM.pdf]

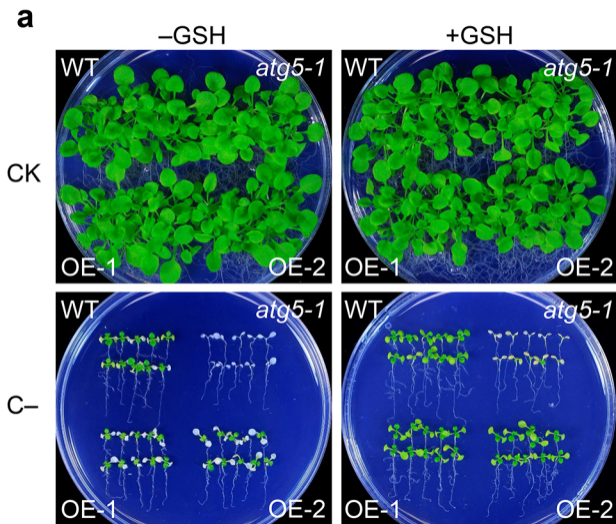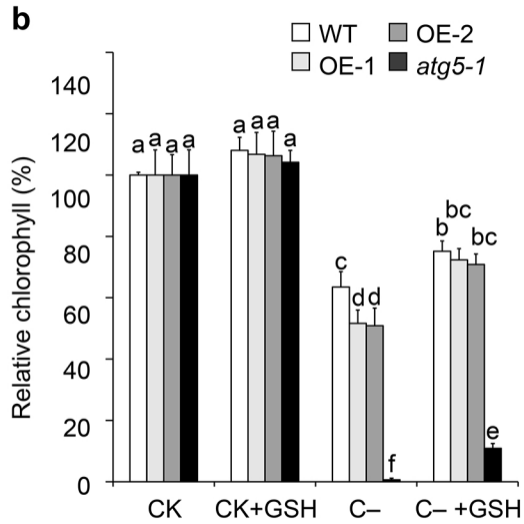

Supplement: Supplementary file 1 — Supplementary Material 1: Fig. S1.Structures of MACP proteins from Arabidopsis. Structures of NSL1 (AT1G28380), MACP1 (AT1G14780), and CAD1 (AT1G29690) were predicted by Alphafold and visualized using PyMOL software. AIM located within the proteins were analyzed by iLIR database. The predicted AIM is shown in red. Fig. S2. The accelerated senescence phenotypes of MACP2-OE plants are dependent on the salicylic acid pathway. a Representative photograph of Wild-type (WT), MACP2-OE-2,eds1-22,pad4, OE-2eds1, OE-2pad4 seedlings in response to nitrogen starvation (N−). For nitrogen starvation treatment, 7-d-old WT, OE-2,eds1-22, pad4, OE-2eds1, and OE-2pad4seedlings grown on half-strength solid MS medium were transferred to N-rich (N+) or N-free (N−) medium and photographed at 5 d into treatment. b Relative chlorophyll contents of 7-d-old wild-type (WT), MACP2-OE-1,eds1-22,pad4,OE-1eds1-22, and OE-1pad4plants seedlings under nitrogen deficiency, expressed as a percentage relative to control plants or seedlings. For each experiment,20 seedlings were used per genotype. All experiments were performed as three biological replicates, each with similar results. Relative chlorophyll contents are means ± SD (n = 3) calculated from three biological replicates. Different lowercase letters indicate significant differences within each group as determined by one-way ANOVA, P < 0.05. Fig. S3. The starvation sensitive phenotypes of MACP2-OE plants are rescued by GSH application. a Wild-type (WT), MACP2-OE (OE-1 and OE-2), and atg5-1 seedlings grown on half-strength solid MS medium for 7 d. The seedlings were transferred to solid sucrose-rich medium (CK) or sucrose-free medium (C−) with or without 500 μM GSH and incubated under normal light/dark conditions or continuous dark conditions for 12 d, respectively, followed by recovery under normal growth conditions for 5 d. b Relative chlorophyll contents of 7-d-old WT, MACP2-OE (OE-1 and OE-2), and atg5-1plants in response to carbon starvatio [file 44307_2025_78_MOESM1_ESM.zip › S3_ESM.pdf]

**a**

WT      OE-2      *eds1-22*      OE *eds1-22*      *pad4*      OE *pad4*

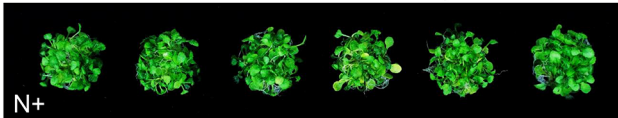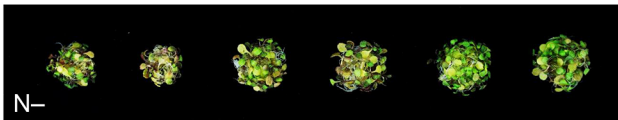**b**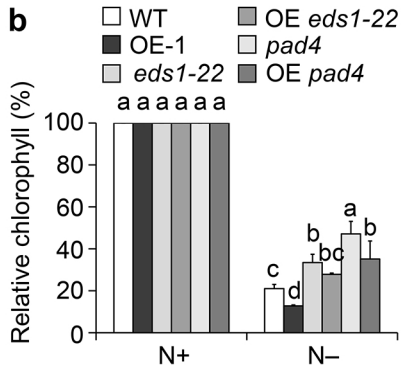

Supplement: Supplementary file 1 — Supplementary Material 1: Fig. S1.Structures of MACP proteins from Arabidopsis. Structures of NSL1 (AT1G28380), MACP1 (AT1G14780), and CAD1 (AT1G29690) were predicted by Alphafold and visualized using PyMOL software. AIM located within the proteins were analyzed by iLIR database. The predicted AIM is shown in red. Fig. S2. The accelerated senescence phenotypes of MACP2-OE plants are dependent on the salicylic acid pathway. a Representative photograph of Wild-type (WT), MACP2-OE-2,eds1-22,pad4, OE-2eds1, OE-2pad4 seedlings in response to nitrogen starvation (N−). For nitrogen starvation treatment, 7-d-old WT, OE-2,eds1-22, pad4, OE-2eds1, and OE-2pad4seedlings grown on half-strength solid MS medium were transferred to N-rich (N+) or N-free (N−) medium and photographed at 5 d into treatment. b Relative chlorophyll contents of 7-d-old wild-type (WT), MACP2-OE-1,eds1-22,pad4,OE-1eds1-22, and OE-1pad4plants seedlings under nitrogen deficiency, expressed as a percentage relative to control plants or seedlings. For each experiment,20 seedlings were used per genotype. All experiments were performed as three biological replicates, each with similar results. Relative chlorophyll contents are means ± SD (n = 3) calculated from three biological replicates. Different lowercase letters indicate significant differences within each group as determined by one-way ANOVA, P < 0.05. Fig. S3. The starvation sensitive phenotypes of MACP2-OE plants are rescued by GSH application. a Wild-type (WT), MACP2-OE (OE-1 and OE-2), and atg5-1 seedlings grown on half-strength solid MS medium for 7 d. The seedlings were transferred to solid sucrose-rich medium (CK) or sucrose-free medium (C−) with or without 500 μM GSH and incubated under normal light/dark conditions or continuous dark conditions for 12 d, respectively, followed by recovery under normal growth conditions for 5 d. b Relative chlorophyll contents of 7-d-old WT, MACP2-OE (OE-1 and OE-2), and atg5-1plants in response to carbon starvatio [file 44307_2025_78_MOESM1_ESM.zip › S2_ESM.pdf]

NSL1

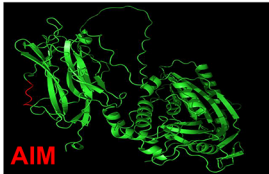

MACP1

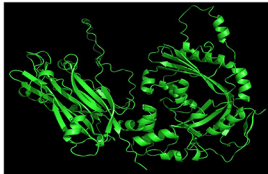

CAD1

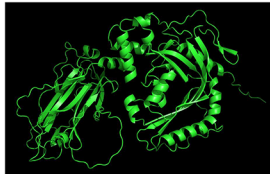

Supplement: Supplementary file 1 — Supplementary Material 1: Fig. S1.Structures of MACP proteins from Arabidopsis. Structures of NSL1 (AT1G28380), MACP1 (AT1G14780), and CAD1 (AT1G29690) were predicted by Alphafold and visualized using PyMOL software. AIM located within the proteins were analyzed by iLIR database. The predicted AIM is shown in red. Fig. S2. The accelerated senescence phenotypes of MACP2-OE plants are dependent on the salicylic acid pathway. a Representative photograph of Wild-type (WT), MACP2-OE-2,eds1-22,pad4, OE-2eds1, OE-2pad4 seedlings in response to nitrogen starvation (N−). For nitrogen starvation treatment, 7-d-old WT, OE-2,eds1-22, pad4, OE-2eds1, and OE-2pad4seedlings grown on half-strength solid MS medium were transferred to N-rich (N+) or N-free (N−) medium and photographed at 5 d into treatment. b Relative chlorophyll contents of 7-d-old wild-type (WT), MACP2-OE-1,eds1-22,pad4,OE-1eds1-22, and OE-1pad4plants seedlings under nitrogen deficiency, expressed as a percentage relative to control plants or seedlings. For each experiment,20 seedlings were used per genotype. All experiments were performed as three biological replicates, each with similar results. Relative chlorophyll contents are means ± SD (n = 3) calculated from three biological replicates. Different lowercase letters indicate significant differences within each group as determined by one-way ANOVA, P < 0.05. Fig. S3. The starvation sensitive phenotypes of MACP2-OE plants are rescued by GSH application. a Wild-type (WT), MACP2-OE (OE-1 and OE-2), and atg5-1 seedlings grown on half-strength solid MS medium for 7 d. The seedlings were transferred to solid sucrose-rich medium (CK) or sucrose-free medium (C−) with or without 500 μM GSH and incubated under normal light/dark conditions or continuous dark conditions for 12 d, respectively, followed by recovery under normal growth conditions for 5 d. b Relative chlorophyll contents of 7-d-old WT, MACP2-OE (OE-1 and OE-2), and atg5-1plants in response to carbon starvatio [file 44307_2025_78_MOESM1_ESM.zip › S1_ESM.pdf]

Fig. 1d

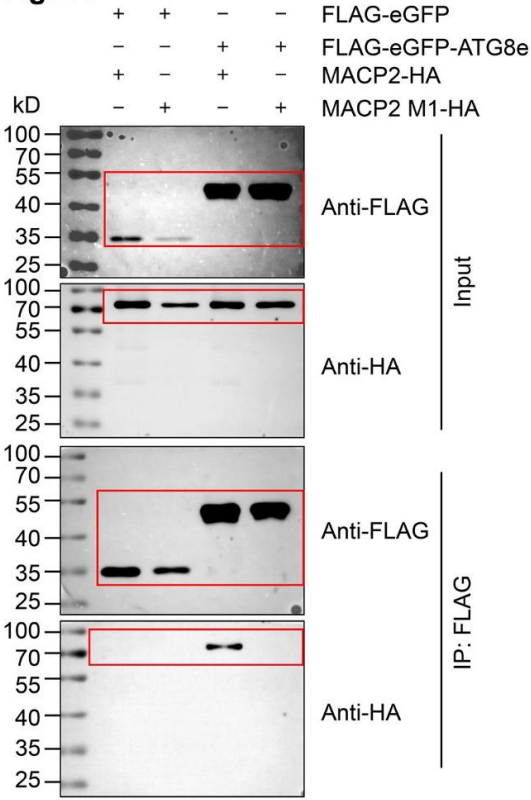

Fig. 7d

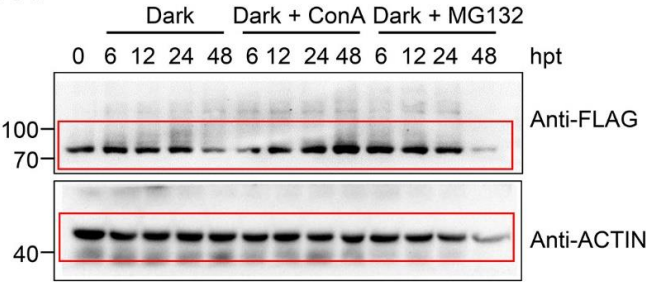

Fig. 7e

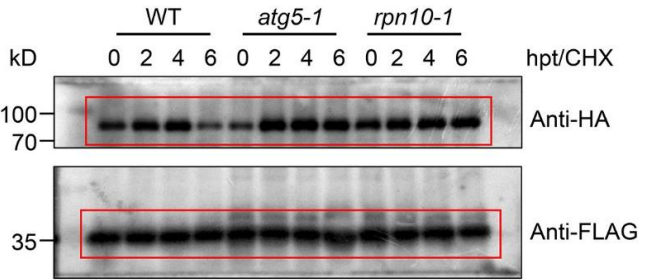

Fig. 6c

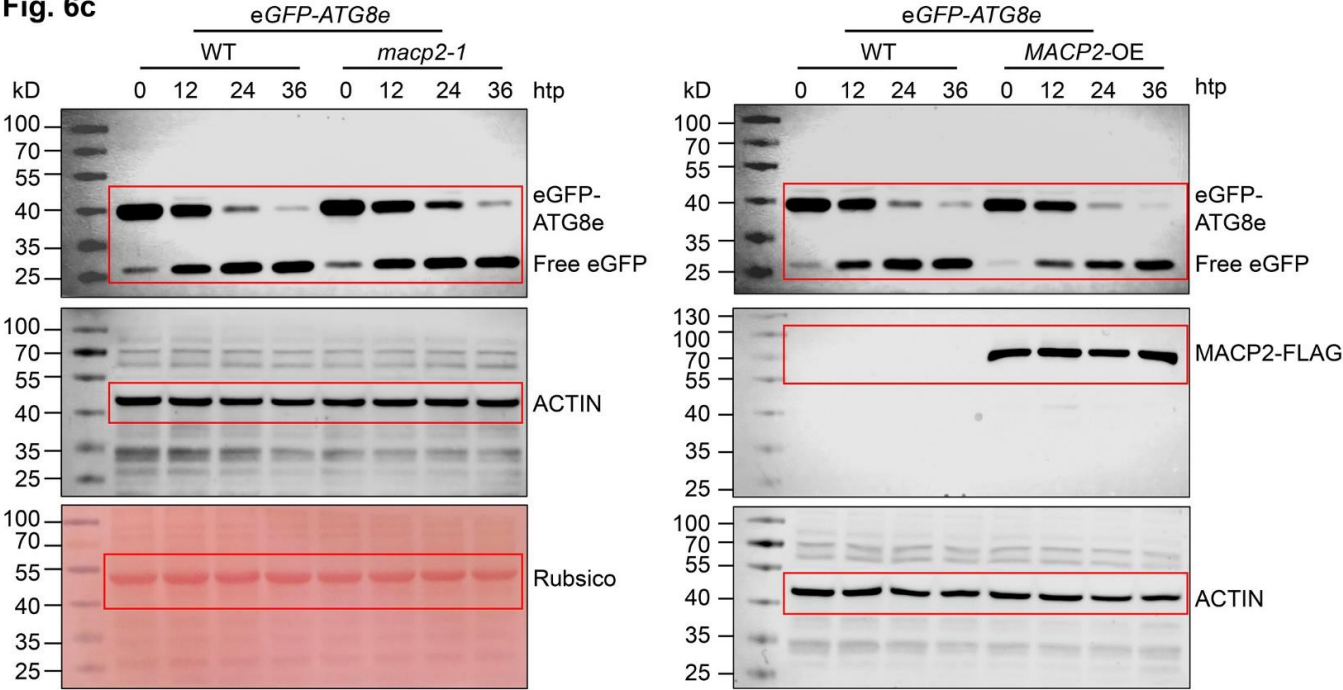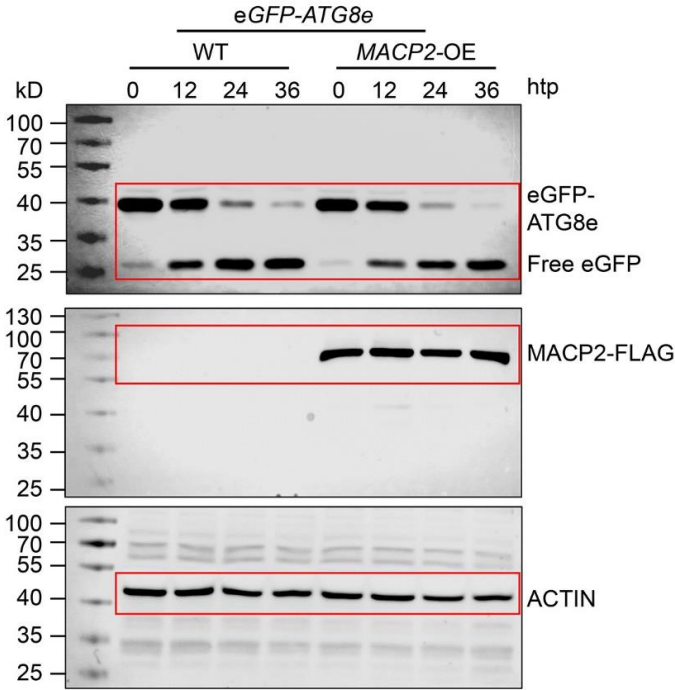

**Fig. S7b**

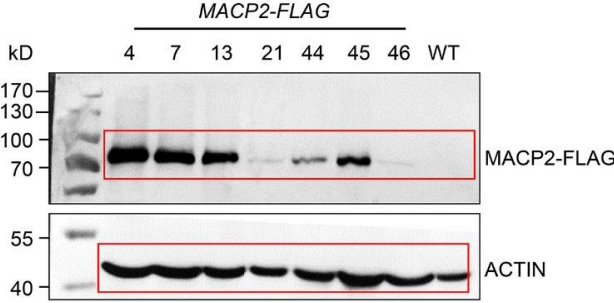

**Fig. S8**

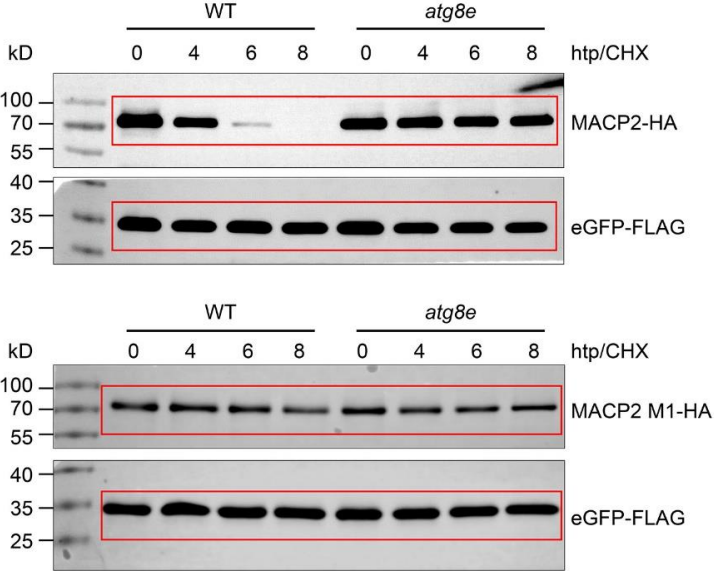

Supplement: Supplementary file 3 — Supplementary Material 3. [file 44307_2025_78_MOESM3_ESM.pdf]
